# Molecular identification of the phosphate transporter family 1 (PHT1) genes and their expression profiles in response to phosphorus deprivation and other abiotic stresses in *Brassica napus*

**DOI:** 10.1371/journal.pone.0220374

**Published:** 2019-07-25

**Authors:** Yu Li, Xue Wang, Hao Zhang, Sheliang Wang, Xiangsheng Ye, Lei Shi, Fangsen Xu, Guangda Ding

**Affiliations:** 1 National Key Laboratory of Crop Genetic Improvement, Huazhong Agricultural University, Wuhan, China; 2 College of Resources and Environment/Microelement Research Center/Key Laboratory of Arable Land Conservation (Middle and Lower Reaches of Yangtze River), Ministry of Agriculture and Rural Affairs, Huazhong Agricultural University, Wuhan, China; INRA, FRANCE

## Abstract

Phosphate (Pi) transporters play critical roles in Pi acquisition and homeostasis. However, little is known about these transporters in oilseed rape. Therefore, the aim of the present study was to characterize the members of the PHT1 gene family in allotetraploid *Brassica napus* and to analyze their expression profiles in response to environmental stresses. In total, 49 PHT1 family members were identified in *B*. *napus*, including 27 genes in the A subgenome and 22 in the C subgenome. Most of the PHT1 proteins were predicted to localize to the plasma membrane. Phylogenetic analysis suggested that the members of the PHT1 gene family can be divided into seven clades, with the introns/exons and protein motifs conserved in each clade. Collinearity analysis revealed that most of the *BnaPHT1* genes shared syntenic relationships with PHT1 members in *Arabidopsis thaliana*, *B*. *rapa*, and *B*. *oleracea*, and that whole-genome duplication (polyploidy) played a major driving force for *BnaPHT1* evolution in addition to segmental duplication. Transcript abundance analysis showed that a broad range of expression patterns of individual *BnaPHT1* genes occurred in response to phosphorus (P) deficiency. In addition, the expression levels of *BnaPHT1* genes can be regulated by different nutrient stresses, including nitrogen (N), potassium (K), sulfur (S) and iron (Fe) stresses. Moveover, salt and drought stresses can regulate the transcript abundances of *BnaPHT1*s, as well as phytohormones including auxin and cytokinin. Gene coexpression analysis based on the RNA-seq data implied that *BnaPHT1*s might cooperate with each other as well as with other genes to regulate nutrient homeostasis in *B*. *napus*. Further analysis of the promoters revealed that GT-1, DRE and P1BS elements are widely distributed within the promoter regions of *BnaPHT1* genes. Our results indicate that *BnaPHT1*s might be involved in cross-talk for sensing the external status of P, N, K, S and Fe, as well as salt and drought stresses. Moreover, these processes might be mediated by phytohormones. Our findings provide the first step in the complex genetic dissection of the Pi transport system in plants and implicate multiple transcriptional regulation, which probably refers to new roles of PHT1 genes in *B*. *napus*.

## Introduction

Phosphorus (P) is one of the important macroelements required for plant growth and development. P is a structural component of nucleic acids and phospholipids, and serves various biological functions in energy metabolism, enzyme activation and signal transduction [1]. There are two different chemical forms of P in soil, as a component of organic compounds and inorganic salts [2]. However, only phosphate (Pi), which is typically present at the micromolar level in soils, can be absorbed directly by plant roots [3]. Several strategies are expressed by plants to enhance Pi acquisition from P-limited soils, such as the remodeling of root morphology, the induction of high-affinity Pi transporters (PTs), and the release of carboxylates and enzymes into the rhizosphere [4]. Since the first report of the high-affinity PT gene *PHO84* in yeast, an increasing number of PTs in various plant species have been identified and functionally characterized [5, 6]. Generally, plant PTs can be grouped into five phylogenetically distinct subfamilies, designated PHT1 to PHT5. Research during the last several decades has shown that the highly conserved PHT1 subfamily of plasma membrane-localized PTs is responsible for Pi uptake from the soil [5, 6].

The plant PHT1 subfamily belongs to the Pi/H^+^ symporter family which is a family member of the large major facilitator superfamily (MFS) [5]. In *Arabidopsis*, there are nine PHT1 members [7]. *AtPHT1;1* and *AtPHT1;4*, which are high-affinity PTs, play major roles in Pi acquisition in both low- and high- P environments [8, 9]. *AtPHT1;8* and *AtPHT1;9* are likely to mediate Pi acquisition by roots only during P starvation conditions [10]. In rice (*Oryza sativa*), 13 PHT1 genes have been identified, and some of them have been functionally characterized, including *OsPHT1;1* [11], *OsPHT1;2* and *OsPHT1;6* [12], *OsPHT1;8* [13]. A very recent study showed that *OsPHT1;3* may function in extremely low-P environments by mediating Pi uptake, translocation and remobilization [14]. Transcription of PHT1 genes can be regulated by transcription factors (TFs) by binding to specific *cis*-acting elements (CAEs) in the promoter region [15]. For example, *AtPHR1*, a member of the MYB superfamily of TEs, can upregulate the expression of *AtPHT1* at low-P concentrations by binding to the CAE named P1BS (GNATATNC) or the P1BS-like element in PHT1 promoters [16]. The plant-specific TF WRKY binds to the W-box CAE (TTGACT/C) in PHT1 promoters and regulates the expression of PHT1 genes [17].

The responses of plants to Pi starvation are initiated by signaling pathways that are shared with responses to other environmental challenges, implying cross-talk occurs between Pi and other abiotic stress signaling pathways [15, 18]. These signaling pathways often include the involvement of phytohormones. For instance, cytokinin (CTK) is implicated in response to Pi starvation, as the lack of CTK receptors shows reduced CTK repression of several Pi starvation-responsive genes such as PHT1 genes [19], while auxin modulates the developmental adaptation of plant roots to Pi starvation [20]. In addition, the transcription of PHT1 genes can be regulated by auxin because their promoters contain auxin-related CAEs such as AuxREs, AuxRR-cores, TGA-elements, and TGA-boxs [15]. Other factors such as drought, salt and nutrient stresses can regulate the transcription of PHT1 genes, possibly via action of phytohormones [15, 21, 22].

Homologs of PHT1s have been identified in various plant species, such as soybean [21], tomato [23], apple [24], wheat [25, 26], sorghum and flax [27]. Although previous studies have reported that *BnPHT1;4* is involved in P uptake, root architecture regulation and seed germination, the roles of the PHT1 family genes in allotetraploid rapeseed (*Brassica napus*) are still unclear [28, 29]. *B*. *napus* (genome AACC, 2n = 38), an important oil crop species, was formed by recent allopolyploidy between ancestors of *B*. *oleracea* (Mediterranean cabbage, genome CC, 2n = 18) and *B*. *rapa* (Asian cabbage or turnip, genome AA, 2n = 20), which resulted in a the genome size of *B*. *napus* being more than six times larger than that of *Arabidopsis thaliana* [30]. However, *B*. *napus* is sensitive to P deficiency. A lack of available P in the soil may inhibit the growth of *B*. *napus*, and its yield production and quality obviously decrease in return [31]. Thus, it is vitally important to characterize the members of the PHT1 family for improved understanding of the functional divergence of PHT1 genes in regulating the growth and development of *B*. *napus*. The aim of this study was therefore to identify and characterize the putative PHT1 family genes based on the published *B*. *napus* genome [30], and to investigate the expression profiles of the *B*. *napus* PHT1 genes in response to various environmental changes. Our works described here offers the first step in the complex genetic dissection of the Pi transport system in *B*. *napus*.

## Materials and methods

### Identification of the PHT1 family genes

The sequences of nine PHT1 family genes obtained from the *Arabidopsis* database in TAIR (https://www.arabidopsis.org/) were used as references to perform BLASTP searches within the CNS-Genoscope genomic database (http://www.genoscope.cns.fr/brassicanapus/) [30]. Redundant sequences were removed manually. The hidden Markov model of the Pfam database (http://pfam.sanger.ac.uk/search), the SMART database (http://smart.embl-heidelberg.de/) and the NCBI Conserved Domain Search database (https://www.ncbi.nlm.nih.gov/Structure/cdd/wrpsb.cgi) were used to confirm that all the genes belonged to the PHT1 family. The genomic DNA, cDNA, CDSs and protein sequences of the PTs were derived from the *B*. *napus* genome database. The *B*. *oleracea* and *B*. *rapa* PHT1 protein sequences were acquired, respectively, from the Phytozome 12.0 database (https://phytozome.jgi.doe.gov/pz/portal.html) and the *Brassica* Database (BRAD, http://brassicadb.org/brad), respectively, using the same method as that described above.

### Characterization of the PHT1 family genes

The molecular weight (MW) and isoelectronic points (PI) were calculated using the ExPASy tool (http://www.expasy.org/tools/). Grand average of hydropathy (GRAVY) values were calculated using the PROTPARAM tool (http://web.expasy.org/protparam). Subcellular localization was predicted by the WoLF PSORT server (https://wolfpsort.hgc.jp/). The structure of the PHT1 family genes was obtained using the GSDS online software (http://gsds.cbi.pku.edu.cn). The MEME tool (http://meme-suite.org/tools/meme) was used to identify potential conserved motifs in *B*. *napus*, *B*. *rapa*, *B*. *oleracea* and *Arabidopsis*, using the following parameter settings for the distribution of motifs: the optimum motif width was 6–50, and the maximum number of motifs was 15. Only motifs with an E-value ≤ 1×10^−10^ were used for further analysis [32].

### Chromosomal location and gene duplication analysis

Physical location information of the *BnaPHT1* genes was retrieved from the CNS-Genoscope genomic database, and were mapped to rapeseed chromosomes using Circos [33]. Gene duplication events and collinearity relationships were analyzed using Multiple Collinearity Scan toolkit (MCScanX) [34]. The criteria for analyzing potential gene duplications were: (a) length of alignable sequence covers >75% of longer gene, and (b) similarity of aligned regions >75%. The syntenic maps were constructed using MCScanX to exhibit the synteny relationship of the orthologous PHT1 genes from *Arabidopsis*, *B*. *napus*, *B*. *rapa* and *B*. *oleracea*.

### Multiple alignment, phylogenetic and evolutionary pressure analysis

Multiple sequence alignments of the PHT1 genes in *Arabidopsis*, *B*. *rapa*, *B*. *oleracea*, and *B*. *napus* were performed using ClustalW. A phylogenetic tree was subsequently constructed using MEGA 5.1 with neighbor joining (NJ) method [35]. The bootstrap value was set at 1000 replications to assess tree reliability. To analyze the evolutionary pressure of the PHT1 proteins, the synonymous (Ks), nonsynonymous (Ka) and Ka/Ks values were calculated based on amino acid and CDS alignments between *Arabidopsis* and *B*. *napus*. Pairwise alignments of gene CDSs without stop codons were performed with ClustalW2 (https://www.ebi.ac.uk/Tools/msa/clustalw2/). The output was submitted to the online program PAL2NAL (http://www.bork.embl.de/pal2nal/index.cgi) to calculate the Ks and Ka substitution rates [36]. In general, a Ka/Ks ratio greater than one means positive selection, whereas a ratio less than one indicates a functional constraint and a ratio equal to one means neutral selection [37].

### Regulatory cis-element analysis in gene promoters

The 2.0-kb upstream sequences of the initiation codon of the *B*. *napus* PHT1 family genes were obtained from the CNS-Genoscope database. Nine CAEs associated with P homeostasis (P1BS and W-box elements), the salt stress response (GT-1 elements and DREs), the drought stress response (MYCR and MBS elements), and the auxin response (TATC-box, TGA and CATATGGMSAVR elements) were identified in this region. The results of the enrichment analysis of the CAEs was displayed by WordArt online software (http://wordart.com/). The position of the CAEs was mapped using IBS online software (http://ibs.biocuckoo.org/online.php).

### Plant materials and growth condition

The rapeseed genotype “Eyou Changjia”, which is a P-efficient genotype, was used for expression analysis [31]. Plants were grown hydroponically in an illuminated culture room with a 16 h light/8 h dark photoperiod at 22 ^o^C, and a light intensity of 300–320 mmol protons m^-2^ s^-1^. Seeds were soaked in deionized water in the dark for two days and subsequently transferred to a net floating on 0.5 mM CaCl_2_ solution for three days. The seedlings were then grown in a modified full-strength Hoagland’s solution (pH 5.8). The solution contained Ca(NO_3_)_2_ 5.0 mM, KNO_3_ 5.0 mM, KH_2_PO_4_ 1.0 mM, MgSO_4_ 2.0 mM, H_3_BO_3_ 46.0 μM, MnCl_2_ 9.0 μM, CuCl_2_ 0.3 μM, ZnCl_2_ 0.8 μM, Na_2_MoO_4_ 0.32 μM and EDTA-iron (Fe) 50.0 μM. After growing for nine days, the plants were treated with various stress conditions for six days, except drought stress, which was applied for three days. For P deficiency treatment, KH_2_PO_4_ was replaced by K_2_SO_4_. For nitrogen (N) deficiency treatment, Ca(NO_3_)_2_ and KNO_3_ were replaced by CaSO_4_ and K_2_SO_4_, respectively. For potassium (K) deficiency treatment, KH_2_PO_4_ and KNO_3_ were replaced by NaH_2_PO_4_ and NaNO_3_, respectively. For sulfur (S) deficiency treatment, MgSO_4_ was replaced by MgCl_2_. For Fe deficiency treatment, EDTA-Fe in Hoagland’s solution was removed. For NaCl stress, 150 mM NaCl was added in Hoagland’s solution. For drought stress, 20% (W/V) PEG6000 was added in Hoagland’s solution. Auxin (IAA) and CTK were added to the Hoagland’s solution for hormone treatments at a final concentration of 100 μM. The roots and fully expanded leaves were sampled separately for RNA extraction at harvest. For each treatment, three replicates were included. For each replicate, four plants were sampled and then mixed together. The experiments were conducted independently at least two times until similar expression results were obtained, and representative data from a single experiment are presented.

### Quantitative real-time PCR analysis

The total RNA of each sample was extracted using a RNeasy Plant Mini Kit (Qiagen), and cDNA was synthesized from First Strand cDNA synthesis kit (Toyobo). The qRT-PCR was carried out in a 10μL volume containing 2μL cDNA, 0.3μL primers, 5μL KAPA SYBR FAST qPCR Kit Master Mix (2X) Universal (KAPA, USA). The thermal cycle was as follow: 95 ^o^C for 5 min; 40cycles of 95 ^o^C for 15 s, 60 ^o^C for 30 s, 72 ^o^C for 20 s. Real-time PCR were performed on the QuantStudio 6 Flex instrument (Life Technologies, USA). The housekeeping gene *EF1-α* (Accession number: DQ312264) was used as an internal standard to normalize the expression level of the target genes. Relative gene expression was calculated with the 2^*-ΔΔCt*^ method. The expression of nine markers were detected (S1 Fig). Gene-specific primers used in this study were listed in S1 Table.

### Coexpression networks of the *BnaPHT1* family genes using RNA-seq data

Gene coexpression network analysis was performed based on the RNA-seq data. For the RNA-seq experiment, 20-day-old plants were treated with P-free nutrient solution for 10 days. Fully expanded leaves (leaf lamine) and roots were then harvested separately for RNA extraction with three biological replicates. A total of 12 RNA samples were subjected to the Illumina HiSeq 2000 platform (Illumina, USA). A total of 643,846,484 raw reads and 571,929,682 clean reads were generated, with an average of 5.0 Gb of sequencing data per sample. The transcript abundance (FPKM value) of each gene was calculated based on the length of the gene and the reads mapped to that gene. The interactions of the target gene sets were retrieved from the STRING protein database (http://string-db.org/), and the weight value of the target gene sets was calculated using the WGCNA R package based on the FPKM values. The gene coexpression networks were visualized by Cytoscape software [38].

### Statistical analysis of data

Statistics was performed by Duncan’s test or Student’s *t* test. Significance of differences was defined as *P < 0.05, ** *P* < 0.01.

## Results

### Identification of the PHT1 family genes in *B*. *napus*

Based on the homology with nine PHT1 family members in *Arabidopsis*, a total of 49 PHT1 genes were identified in the whole genome of *B*. *napus*, including 27 genes located in the A subgenome and 22 in the C subgenome. These genes were named *BnaPT1* to *BnaPT49* (S2 Table). The same approach was used again and led to the identification of 28 and 23 PHT1 genes in *B*. *rapa* and *B*. *oleracea* (S3 Table), respectively. These genes were named *BrPT1* to *BrPT28* in *B*. *rapa* and *BoPT1* to *BoPT23* in *B*. *oleracea* (S2 Fig). To compare the evolutionary diversity of the PHT1 family members in the Viridiplantae, we investigated the copy number variation (CNV) in 24 plant species. The results showed that the copy number of PHT1 family members in *B*. *napus*, *B*. *rapa* and *B*. *oleracea* was the largest among the 24 species, especially in the allotetraploid *B*. *napus* (S3 Table). This finding could be attributed to different intrinsic P requirements for plants during evolution. Characteristic analysis revealed that the length of genomic DNA without the untranslated region varied from 1530 bp to 4850 bp, while the protein length of BnaPHT1s ranged from 433 aa to 565 aa (S2 Table). MW, which is related to protein length, ranged from 47.72 kDa to 62.09 kDa, and the pI of the PHT1 proteins ranged from 6.09 to 9.33, 44 of which were greater than seven. The GRAVY value, which was defined by the sum of the hydropathy values of all amino acids divided by the protein length, was positive and varied from 0.30 to 0.43, indicating that the *BnaPHT1* proteins could be hydrophobic. The subcellular localization predicted by WoLF PSORT showed that most of the *BnaPHT1* proteins were located on the plasma membrane, except *BnaPT4*, *BnaPT31*, and *BnaPT45* which were located on the vacuolar membrane, and *BnaPT10*, which was located on the plasma membrane or the vacuolar membrane (S2 Table).

### Phylogenetic analysis, gene structure and conserved motif analysis of the *BnaPHT1* genes

**Fig 1:**
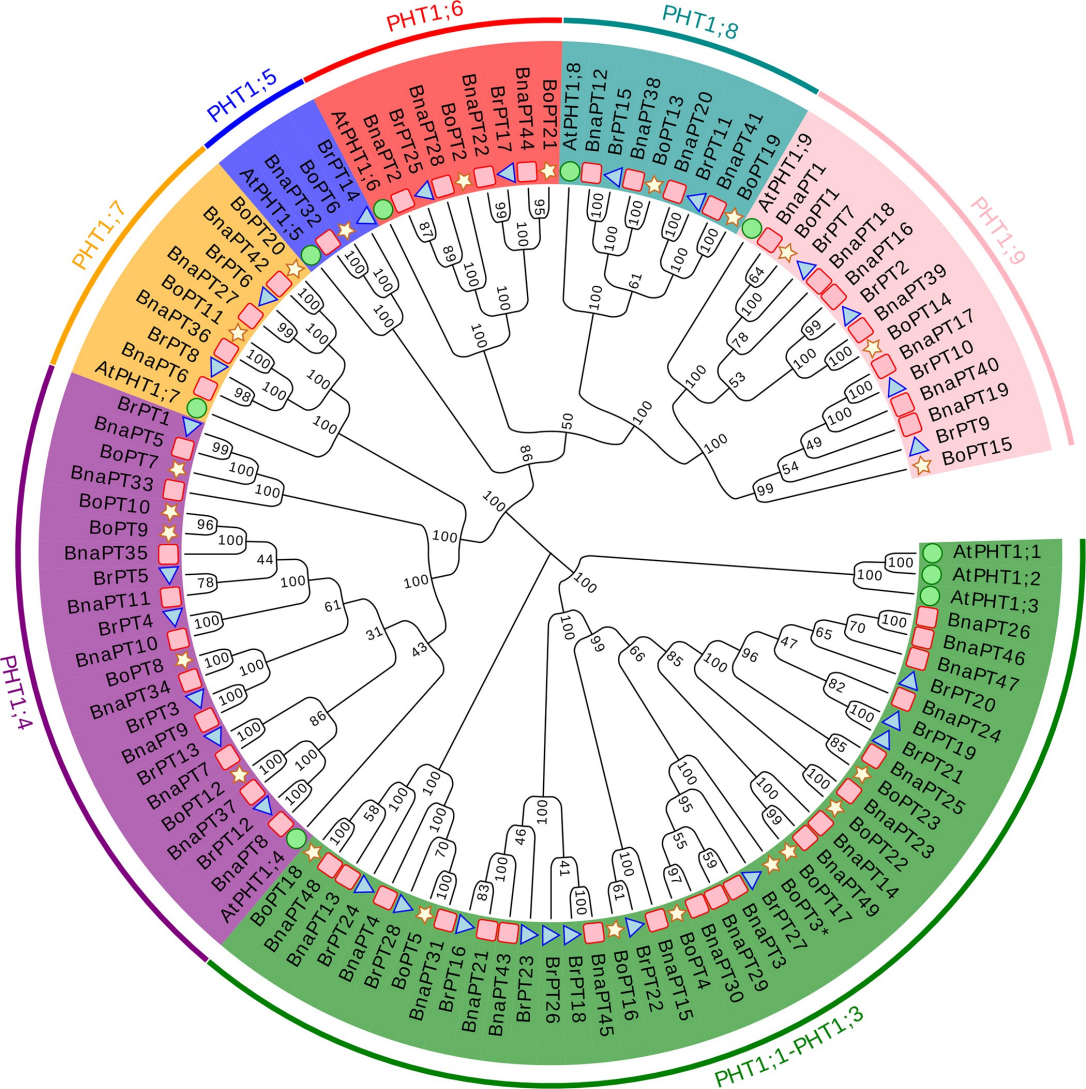
Phylogenetic tree of coding nucleotide sequences of the phosphate transporter family 1 (PHT1) in *Brassica napus*, *B*. *oleracea*, *B*. *rapa* and *Arabidopsis thaliana*. The phylogenetic tree was constructed by MEGA 5.1 with neighbor-joining method and 1000 replicates. The analysis involved 109 nucleotide sequences including 49 from *B*. *napus* (pink red rectangle), 28 from *B*. *rapa* (light blue triangle), 23 from *B*. *oleracea* (light yellow star) and nine from *Arabidopsis* (light green circle).

To determine the evolutionary relationships of the members of the *BnaPHT1* gene family genes with those of the *B*. *napus* ancestor species, we constructed a phylogenetic tree comprising 109 PHT1 proteins, from *B*. *napus* (49), *B*. *rapa* (28), *B*. *oleracea* (23) and *Arabidopsis* (9) based on a multialignment via MEGA 5.1. Our results showed that all the PHT1 homologs could be classified into seven clades. *AtPHT1;1*, *AtPHT1;2* and *AtPHT1;3*, which are highly similar to each other, were classified as Clade 1 together with 19, 11 and eight PHT1 genes in *B*. *napus*, *B*. *rapa* and *B*. *oleracea*, respectively. The remaining subfamilies (Clade 2 to Clade 7) were divided in accordance with the left six PHT1 members (*AtPHT1;4* to *AtPHT1;9*) in *Arabidopsis*. In addition, almost all of the *B*. *napus* PHT1 genes appeared as pairs with the PHT1 members in *B*. *rapa* or *B*. *oleracea* in terms of phylogenetic relationships (Fig 1, S3 Fig).

We further analyzed the gene structure of the 109 PHT1s in *B*. *napus*, *B*. *rapa*, *B*. *oleracea* and *Arabidopsis* (S3 Fig). The results showed that most of the genes in Clade 1, Clade 6 and Clade 7 contained one intron, except *BrPT19*, *BrPT21*, *BoPT14*, *BoPT19* and *BoPT23*, which contained two introns, while all the genes in Clade 2, Clade 3 and Clade 5 had no introns, with the exception of *BrPT5*, which contained one intron. The genes in Clade 4 possessed two introns. Gene structure analysis of the PHT1 family genes in the four species indicated that the gene structure differed across the different subgroups but it was conserved in the same subgroup. In addition, the amino acids of the PHT1 genes were submitted to MEME for domain and motif structure analyses. In total, 15 conserved motifs were identified within the 109 PHT1 genes, while the conserved PHT1 signature (GGDYPLSATIMSE) was found in motif 3 (S4 Fig). Moreover, most of the closely related genes in each subgroup shared a similar motif composition, but it varied largely among different subfamilies. The similar motif arrangements among PHT1 proteins within subgroups indicated that the protein architecture was conserved within a specific subfamily. Overall, the similar gene structure and conserved motif composition of the PHT1 members in the same group, together with the results of the phylogenetic analysis, strongly support the reliability of the group classification.

### Chromosomal distribution and duplication of the *BnaPHT1* genes

Chromosomal location analysis revealed that 47 *BnaPHT1*s were distributed unevenly on 15 chromosomes, except for A01, A10, C01 and C07, with 27 in the A subgenome and 22 in the C subgenome (S2 Fig). Two members (*BnaPT48* and *BnaPT49*) were located in the C subgenome but could not be mapped to a specific chromosome. Some chromosomes (e.g., ChrA09) had relatively many genes, whereas others had relatively few (e.g., ChrA03). Chromosome A09 contained the greatest number of *BnaPHT1* genes, and the six *BnaPHT1* genes that were located on chromosome A09 appeared in a gene cluster. There was no positive correlation between chromosome length and number of PHT1 genes. In addition, both *BrPHT1*s and *BoPHT1*s were both mapped onto eight chromosomes, except ChrA01 in *B*. *rapa* and ChrC01 in *B*. *oleracea* (S2 Fig).

**Fig 2:**
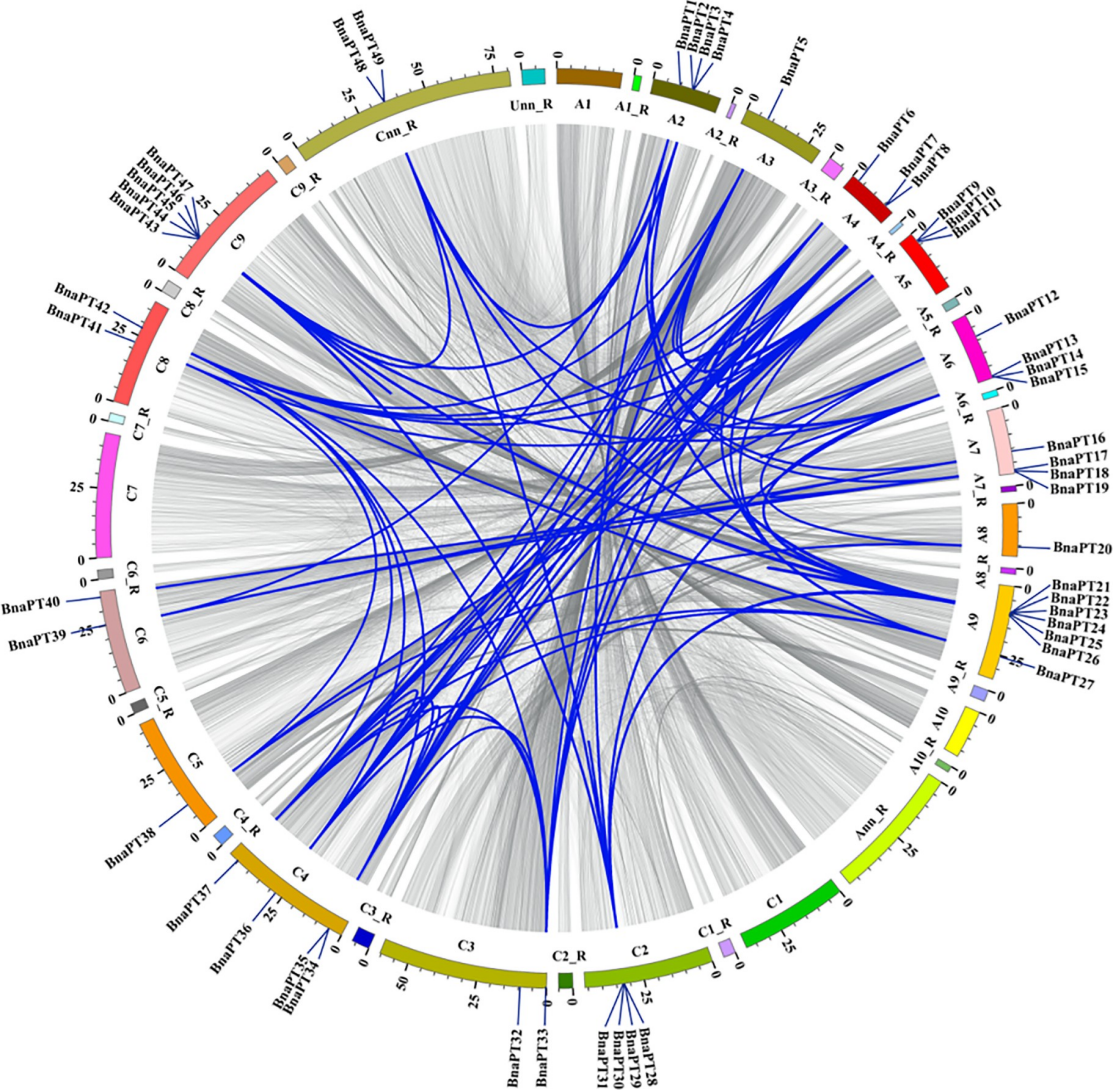
Schematic representations for the chromosomal distribution and interchromosomal relationships of rapeseed PHT1 genes. Gray lines indicate all syntenic blocks in the *Brassica napus* genome, and the blue lines indicate syntenic PHT1 gene pairs. The chromosome number is indicated at the bottom of each chromosome. R, random.

**Fig 3:**
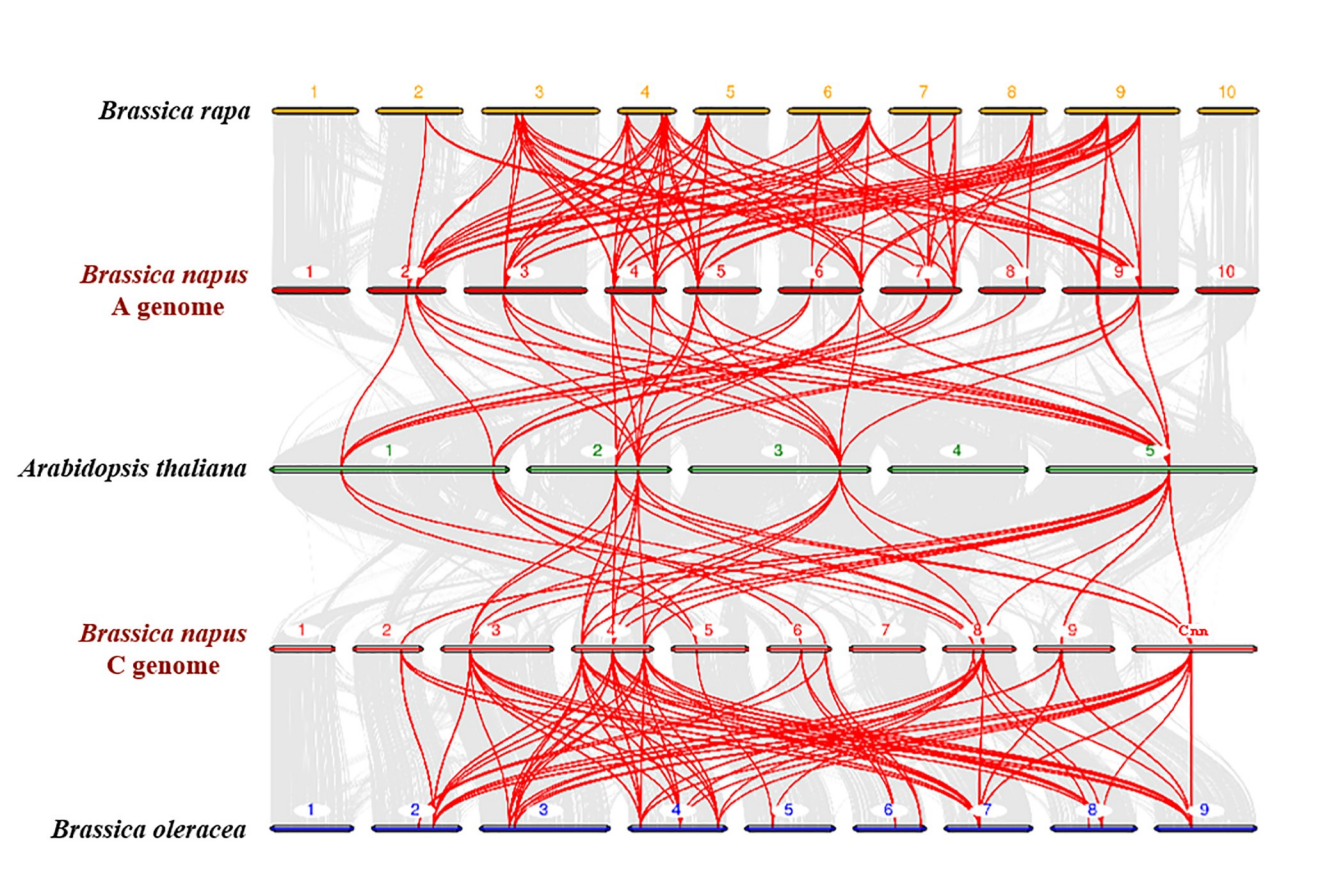
Synteny analysis of PHT1 genes in *Brassica napus*, *B*. *rapa*, *B*. *oleracea* and *Arabidopsis thaliana* chromosomes. Gray lines in the background indicate the collinear blocks within *B*. *napus* and other plant genomes, while the red lines highlight the syntenic PHT1 gene pairs. Genes located on *B*. *napus* A genome are syntenic with genes of *B*. *rapa* and *A*. *thaliana*, while genes located on *B*. *napus* C genome are syntenic with genes of *B*. *oleracea* and *A*. *thaliana*.

A chromosomal region within 200 kb containing two or more homologous genes is defined as a tandem duplication event [39]. In this study, we found that 17 *BnaPHT1* genes (34.7%) were clustered into seven tandem duplication event regions on chromosomes A05, A06, A07, A09, C02, C04 and C09 (S4 Table). The number of duplicated genes in tandem clusters ranged from 2–4. In addition to the tandem duplication events, we also identified 195 segmental duplication events with 48 PHT1 genes by BLASTP and MCScanX methods (Fig 2). These results indicated that segmental duplication events played a major driving force for the expansion of the *BnaPHT1* family. In the present study, we also identified 28 and 23 PHT1 genes in the *B*. *rapa* and *B*. *oleracea* genomes (S2 Fig), respectively. To further infer the phylogenetic mechanisms of the *B*. *napus* PHT1 family, we constructed a comparative syntenic map of *B*. *napus* and its ancestors (*Arabidopsis*, *B*. *rapa* and *B*. *oleracea*). Collinearity analysis revealed that there were strong orthologs of PHT1 genes between *B*. *napus* and the other three ancestral species (Fig 3). Twenty-three and 20 of the genes in the A subgenome of *B*. *napus* showed syntenic relationships with 26 and eight PHT1 genes in the *B*. *rapa* and *Arabidopsis* genomes, respectively. In contrast, 20 and 19 of the genes in the *B*. *napus* C subgenome were syntenic with 21 and eight of the *B*. *oleracea* and *Arabidopsis* genomes, respectively. The fact that nearly all of the homologous *BrPHT1*s and *BoPHT1*s maintained a syntenic relationship with *BnaPHT1*s suggested that whole-genome duplication (polyploidy) also played a major driving force for *BnaPHT1* evolution in addition to segmental duplication.

To better understand the evolutionary constraints acting on the PHT1 gene family, the Ka, Ks and Ka/Ks ratio were estimated for *B*. *napus*. Our results showed that all the segmental and tandem-duplicated *BnaPHT1* gene pairs had a Ka/Ks ratio of less than one (S5 Fig), indicating that the PHT1 family in *B*. *napus* might have experienced strong purifying selective pressure during evolution.

### Differential expression profiles of the *BnaPHT1* genes under various environments

**Fig 4:**
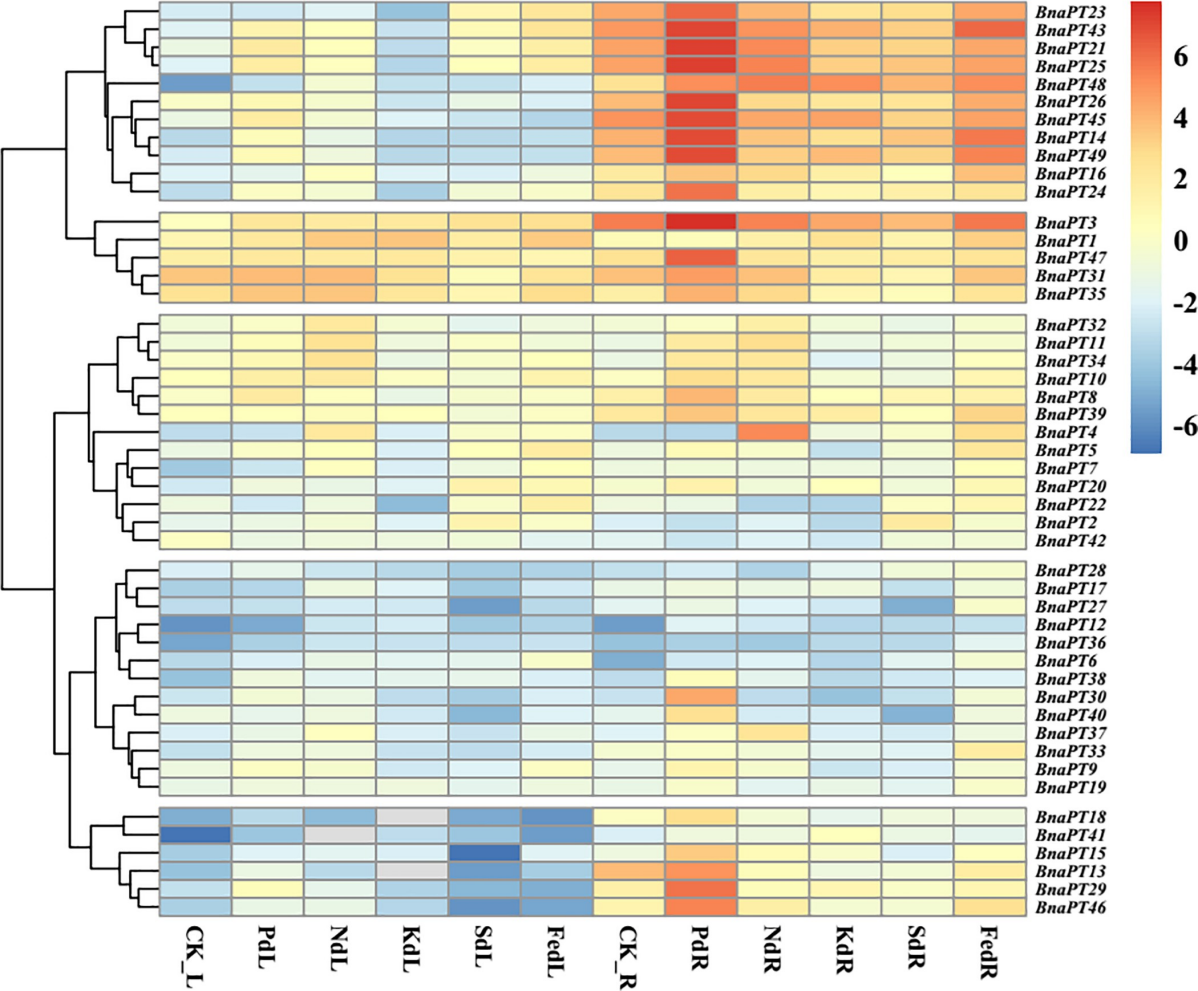
Expression profiles of the PHT1 family genes in the leaves and roots of *Brassica napus* under various nutrient stress conditions. Seedlings of 14 days old were exposed to various nutrient deficiency environments for six days. The fully expanded leaf and roots were sampled separately for RNA extraction. L, leaf. R, roots. CK, full strength Hoagland’s solution. Pd, phosphorus deficiency. Nd, nitrogen deficiency. Kd, potassium deficiency. Sd, sulfur deficiency. Fed, iron deficiency. The color scale is shown on the right side. Gray box indicates data undetected. Heat map of gene expression profiles was generated using pheatmap package in R after data normalization.

**Fig 5:**
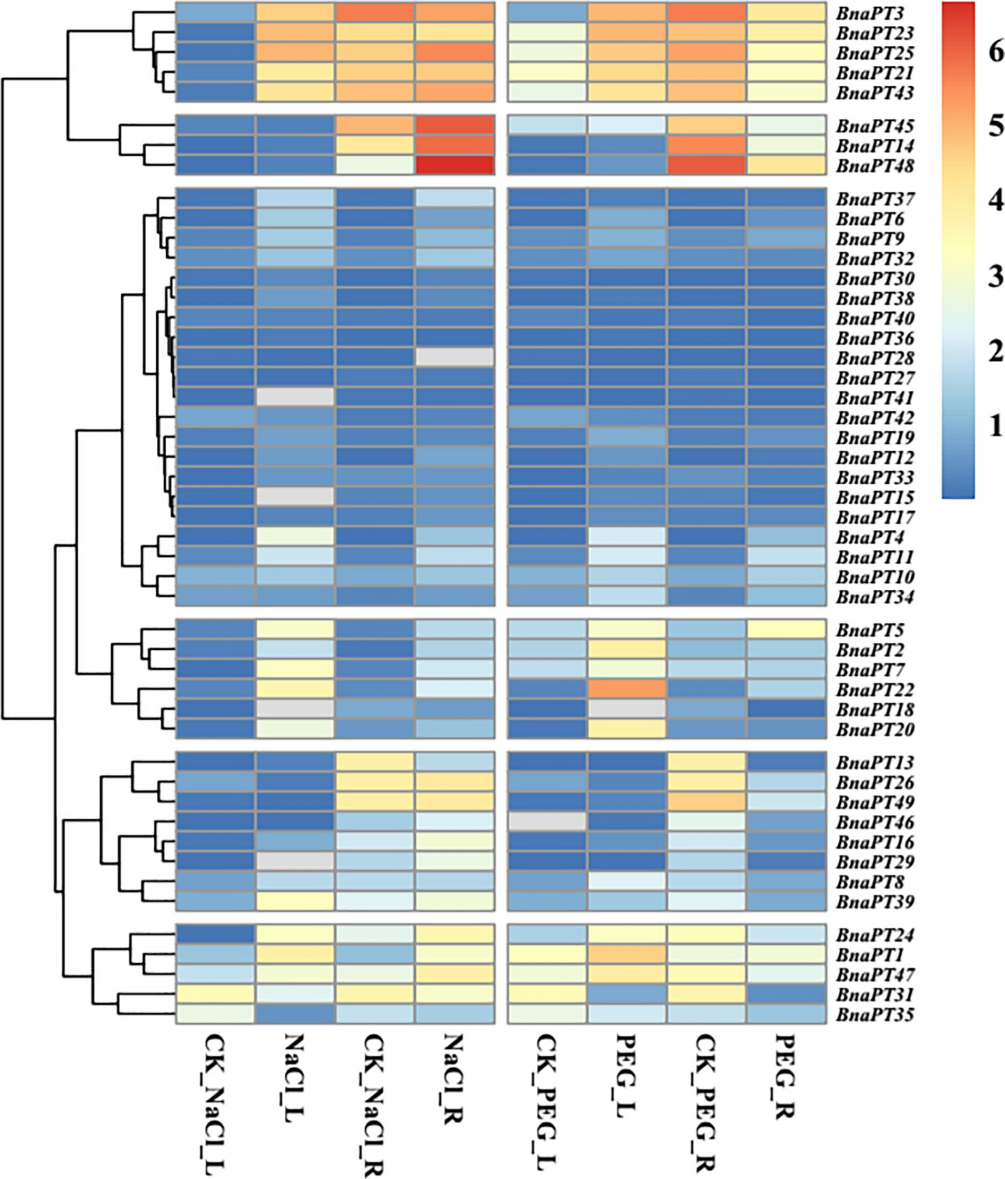
Expression profiles of the PHT1 family genes in the leaves and roots of *Brassica napus* under salt and drought stresses. For salt stress analysis, 14-d-old seedlings were treated with Hoagland’s solution containing 150mM NaCl for six days. For drought stress analysis, seedlings of 14 days old were treated with Hoagland’s solution containing 20% (W/V) PEG for three days. The full expanded leaf and roots were sampled separately for RNA extraction at harvest. L, leaf. R, roots. CK, full strength Hoagland’s solution. NaCl, salt stress. The color scale is shown on the right side. Gray box indicates data undetected. Heat map of gene expression profiles was generated using pheatmap package in R after data normalization.

**Fig 6:**
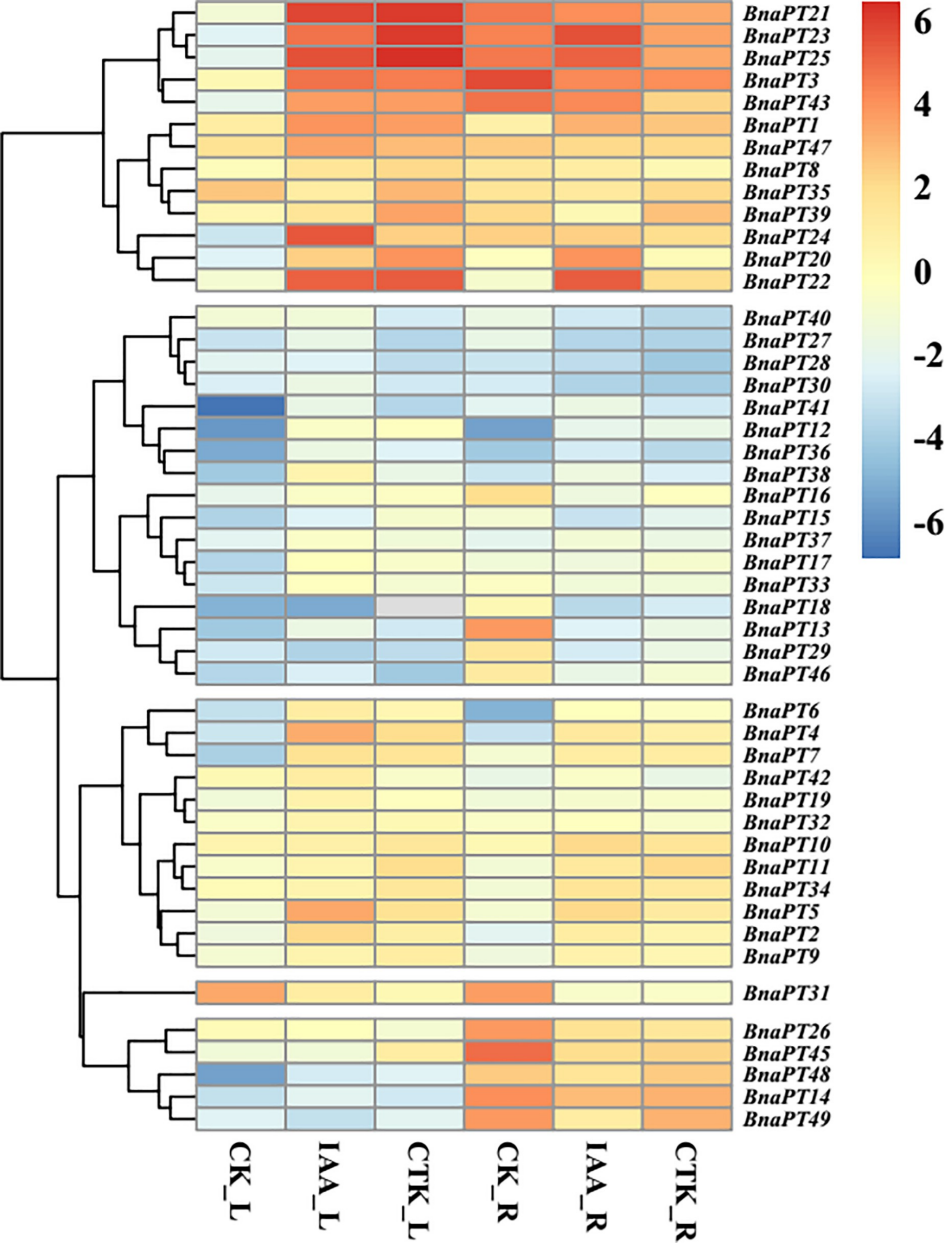
Expression profiles of the PHT1 family genes in leaves and roots of *Brassica napus* under auxin (IAA) and cytokinin (CTK) treatments. Seedlings of 14 days old were treated with Hoagland’s solution containing 100 μM IAA and 100 μM CTK for six days, respectively. The fully expanded leaf and roots were sampled separately for RNA extraction at harvest. L, leaf. R, roots. CK, full strength Hoagland’s solution. The color scale is shown on the right side. Gray box indicates data undetected. Heat map of gene expression profiles was generated using pheatmap package in R after data normalization.

To date, little is known about the expression profiles of the *BnaPHT1* genes in response to environmental changes, which may elucidate their functions in detail. In the present study, the transcriptional levels of 48 *BnaPHT1* genes were examined systemically by qRT-PCR under various stress conditions, except *BnaPT44*, which was not detected (Figs 4–6, S6 Fig). Generally, the accumulation of PTs was associated with both tissues and contrasting environment treatments (S6 Fig). Some of the PHT1 genes were predominantly detected in the roots and specifically and strongly induced by P deficiency, while others responded to hormone treatments.

The expression levels of *BnaPHT1*s in response to P, N, K, S and Fe deficiencies were clustered and divided into five groups (Fig 4). Under P starvation conditions, the expression of nine genes was significantly upregulated, and only one gene (*BnaPT22*) was significantly downregulated in leaves, while approximately 80% (38/49) of the *BnaPHT1*s were upregulated by P deprivation in the roots. N starvation significantly enhanced the expression of *BnaPT1*, *BnaPT3*, *BnaPT4*, *BnaPT6*, *BnaPT7*, *BnaPT9*, *BnaPT11*, *BnaPT12* and *BnaPT33*, and significantly inhibited the expression of *BnaPT13* and *BnaPT35* in the leaves, while in the roots, 10 genes were induced by N stress. K deficiency significantly enhanced the expression of *BnaPT1*, *BnaPT3*, *BnaPT7*, *BnaPT12*, *BnaPT17*, *BnaPT38* and *BnaPT43* and inhibited the expression of *BnaPT9*, *BnaPT13*, *BnaPT21*, *BnaPT22*, *BnaPT23*, *BnaPT31*, *BnaPT34* and *BnaPT40* in the leaves. In the roots, two genes (*BnaPT41* and *BnaPT48*) were upregulated by K stress, while seven genes were downregulated. Under S-limited conditions, seven and nine genes were induced and repressed in the leaves, respectively. In the roots, two and 10 genes were up- and down- regulated, respectively. In addition, 16 genes were upregulated and four genes were downregulated by Fe deficiency in the leaves. Among the 20 differentially expressed genes in response to Fe deprivation in the roots, only one (*BnaPT21*) was downregulated, while the others were all upregulated. Interestingly, the expression of *BnaPT48* in the roots increased across all the nutrient-deficient conditions. Forty-two of 49 genes were influenced by at least two types of mineral nutrient deficiencies in the leaves or roots.

Totals of 24 and 19 genes were influenced by salt stress in the leaves and roots, respectively (Fig 5). In the leaves, 21 genes were upregulated, and only three genes (*BnaPT13*, *BnaPT31* and *BnaPT35*) were downregulated. In the roots, all 19 deferentially expressed PHT1 genes were enhanced by salt stress, including *BnaPT48*, which was induced by all the nutrient stresses. In total, 18 genes were upregulated in the leaves under drought stress conditions, and no genes were downregulated. In contrast, among 14 deferentially expressed PHT1 genes in the roots, most of them (11) were downregulated by drought stress, and only three genes (*BnaPT11*, *BnaPT34* and *BnaPT38*) were upregulated (Fig 5).

The hierarchical clusters of expression changes in the *BnaPHT1*s after hormone (CTK and IAA) treatments are displayed in Fig 6. The results showed that the transcription levels of the PHT1 family genes in *B*. *napus* were influenced by CTK and IAA. In total, 24 *BnaPHT1*s were significantly upregulated or downregulated in the leaves by IAA, while in the roots, 23 *BnaPHT1*s were differentially upregulated or downregulated by IAA. Under CTK treatment, 14 and three genes were up and down regulated in the leaves, respectively. In the roots, eight genes were significantly upregulated by CTK, and 10 genes were significantly downregulated by CTK. In addition, three genes (*BnaPT3*, *BnaPT21*, and *BnaPT47*) were significantly induced in leaves and suppressed in the roots in response to CTK. Some genes can be influenced by both IAA and CTK in the leaves and roots, such as *BnaPT11*, *BnaPT12* and *BnaPT31*.

### Coexpression networks of the PHT1 family genes in *B*. *napus*

**Fig 7:**
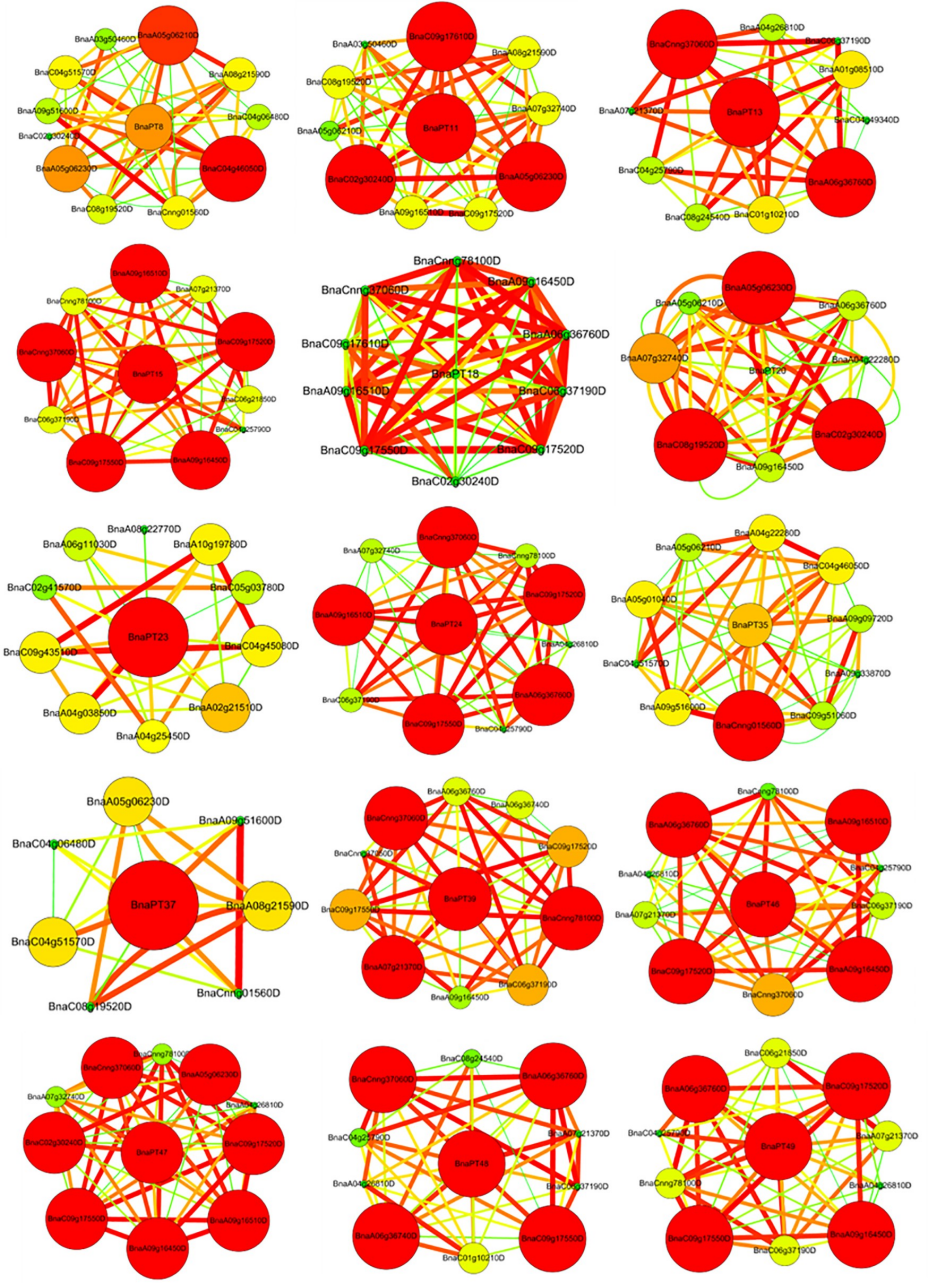
Coexpression networks of 15 PHT1 family genes in *Brassica napus*. Cycle nodes represent genes, and the size of the nodes represents the power of the interrelation among the nodes by degree value. The width of the lines between two nodes represent interactions between genes. The PHT1 family genes located in the center of the network, while the 10 most coexpressed genes were displayed in each network.

To further unravel the coexpression relationships between PHT1 family genes and other genes, we calculated the interaction weight values of the target gene sets based on the FPKM values from the RNA-seq data. Ten sets of 15 *B*. *napus* PHT1 genes with the strongest interactions are shown in Fig 7. Generally, among the 10 strongest interactions, *BnaPT20* interacted with eight PHT1 family genes, while *BnaPT8*, *BnaPT11* and *BnaPT39* interacted with seven PHT1 genes. Both *BnaPT18* and *BnaPT47* were coexpressed with six PHT1 genes. With respect to *BnaPT15*, *BnaPT48* and *BnaPT49*, each was highly corelated with the other five PHT1 genes. Among the 10 strongest interactions, four PHT1 family genes were coexpressed with *BnaPT4*, *BnaPT37* and *BnaPT46*, and three were coexpressed with *BnaPT13* and *BnaPT35*. Only *BnaPT23* interacted with ten genes that do not belong to the PHT1 family. These results indicated that *B*. *napus* PHT1 family members may function together in P homeostasis. Except for PHT1 genes, other genes also exhibit strong interactions with the 15 PHT1 genes, including genes responding to low temperature and salt stresses, PPa (pyrophosphorylase) family members, which may be involved in inorganic diphosphatase activity and Pi metabolic process, ABC family genes, which have ATPase activity, and CBL1 family genes, which are involved in calcium ion binding (Fig 7).

### Identification of the cis-acting regulatory elements

**Fig 8:**
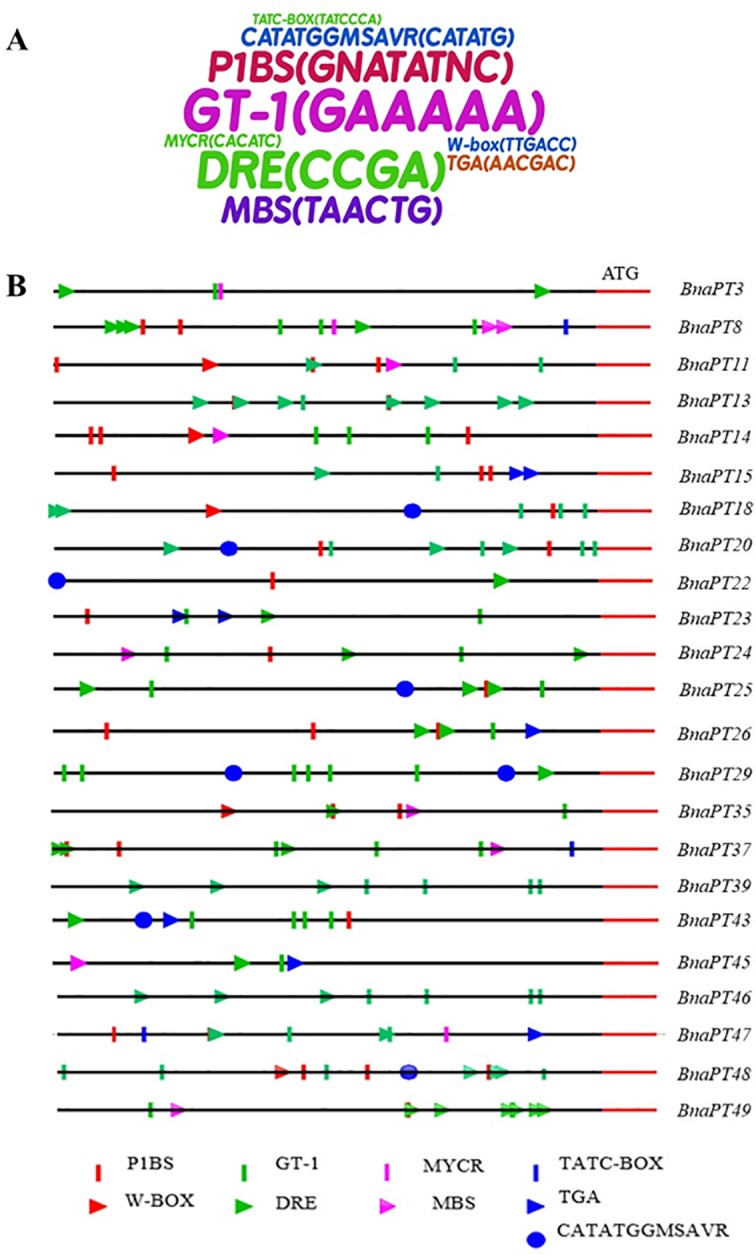
*Cis*-elements in the 2.0-kb promoter regions of the PHT1 family genes in *Brassica napus*. (A) Enrichment of the *cis*-elements in the promoter sequences of *BnaPT*s. The size of the sequence represents the frequency of the *cis*-elements in the promoter regions. (B) Distribution of nine *cis*-elements in the promoters of 23 PHT1 family members. These *cis*-elements are associated with P homeostasis (P1BS and W-BOX), salt stress response (GT-1 and DRE), drought stress response (MYCR and MBS), and auxin response (TATC-BOX, TGA and CATATGGMSAVR).

To understand the potential transcriptional regulation of *BnaPHT1*s, we conducted an *in silico* analysis based on the DNA sequences of the promoter regions. The 2.0-kb upstream region of the initiation codon was used to identify nine CAEs associated with P homeostasis, the salt stress response, the drought stress response and the auxin response (Fig 8, S5 Table). In total, 406 *cis*-elements were identified in the promoters of 49 *BnaPHT1*s, and more than one type of CAEs was identified for each *B*. *napus* PHT1 gene, except *BnaPT21* (S5 Table). These results indicated that complex regulatory networks may be implicated in the transcriptional regulation of *BnaPHT1*s. Among the nine types of CAEs, GT-1, DRE and P1BS were the top three enriched elements (Fig 8A). Approximately one to three P1BS or W-box elements that were involved in P homeostasis existed in the promoters of *BnaPHT1*s, except for seven genes. Forty-eight *BnaPHT1*s contained one to 12 salt-stress-responsive *cis*-elements. In contrast, approximately half of the *BnaPHT1*s contained CAEs associated with drought stress and auxin (Fig 8B, S5 Table).

## Discussion

### High copy number variation of PHT1 family genes in *Brasscia* species

As an essential, nonsubstitutable element for plant growth, P plays a wide range of structural and biological roles [1]. The inorganic Pi concentration in plant tissues is about 5–20 mM; however, in soils, the available P is typically less than 10 μM [5]. This sharp concentration gradient between the plant and the soil indicates the indispensable roles of PTs, which can regulate Pi absorption. Among the five PT families, PHT1 family members are the most reported due to their presence in the plant plasma membrane and their function in Pi acquisition from the soil [5, 6]. Since the first PHT1 gene was cloned in *Arabidopsis* in 1996 [40], an increasing number of PHT1 genes have been identified based on protein sequence identity and conserved signature analyses [6]. However, little is known about the PHT1 family in *B*. *napus*, *B*. *rapa* and *B*. *oleracea*. In this research, we summarized the CNV of the PHT1 family genes in Viridiplantae. Among the 24 species, the CNV of the PHT1 genes varies from five to 49. Interestingly, the highest CNV (49) was detected in *B*. *napus*, followed by *B*. *rapa* (28) and *B*. *oleracea* (23), respectively. The PHT1 family is larger in *B*. *napus* than in any other plant species reported thus far (S3 Table). Compared with *A*. *thaliana*, *Brassica* species experienced an extra whole-genome triplication event that contributed to a gene-level evolution and drove the diversification of the *Brassica* plants. Thus, one *Arabidopsis* gene should theoretically correspond to three orthologs in *B*. *rapa* and *B*. *oleracea*, while *B*. *napus* should contain six syntenic copies of each *Arabidopsis* gene because it was derived from the recent hybridization between *B*. *rapa* and *B*. *oleracea* [30]. In this study, the expansion of PHT1 genes in *Arabidopsis* led to more than five times more gene numbers in *B*. *napus* and two to three times more gene numbers in *B*. *rapa* and *B*. *oleracea* (S3 Table). The homologous synteny and chromosomal location analyses indicated that the *BnaPHT1* genes in the An and Cn subgenomes of *B*. *napus* are closely phylogenetically related to the PHT1 genes in *B*. *rapa* and *B*. *oleracea*, respectively (Figs 1 and 3, S2 Fig). However, duplicated genes might have been lost during evolution, as the synteny between the PHT1 genes of *B*. *napus* and their homologs in *Arabidopsis*, *B*. *rapa* and *B*. *oleracea* was less than expected (Fig 3, S3 Table). Gene duplication is considered one of the primary driving forces in the evolution of genomes and genetic systems. Of all the duplication patterns, segmental and tandem duplications have been purported to be the two main causes of gene family expansion in plants [41]. In the current research, 43 of the 49 *BnaPHT1*s (87.8%) in the *B*. *napus* genome had a syntenic relationship with *BrPHT1*s (23 genes) and *BoPHT1*s (20 genes), as expected. These results indicate that allotetraploidy has contributed largely to the rapid expansion of the PHT1 gene family in *B*. *napus*. Moreover, 195 segmental duplication events were detected to contain 48 of the 49 *BnaPHT1* genes, while only nine tandem duplication events were identified (Fig 2, S4 Table). Taken together, our results indicate that segmental duplication and whole-genome duplication (polyploidy) are the main force for the expansion of the PHT1 gene family in *B*. *napus*. A relatively large number of members within a family suggests successful expansion and rearrangement of the genome by extensive duplication that occurred frequently during evolution [42]. However, the PHT1 genes may have undergone functional divergence during evolution, as indicated by the expression profile analysis (S6 Fig). Thus, additional experiments should be conducted to unravel the functions of *BnaPHT1*s using yeast mutant and transgenic approaches.

Except for BnaPT38, the PHT1 proteins in *B*. *napus* have 509 to 565 amino acids (S2 Table), making them similar in size to AtPHT1 proteins in *Arabidopsis* [5]. Genome polyploidizations is typically accompanied by massive chromosomal rearrangements [43]. In *B*. *napus*, PHT1 genes are dispersed across 15 chromosomes, but the dispersion differed between the A subgenome of *B*. *napus* and *B*. *rapa* and between the C subgenome of *B*. *napus* and *B*. *oleracea* (S2 Fig), indicating that large diversification and chromosomal rearrangements occurred in *Brassica* species during allopolyploidization and domestication. Structural analysis is a powerful method that can be used to obtain valuable information concerning duplication events and phylogenetic relationships of genes within a gene family. In this research, we observed that the PHT1 genes in three *Brassica* species had the same number of exons (1–2) as did those in *Arabidopsis* and rice [5, 42], and the gene structure differed in different clades but was conserved within the same clades (S3 Fig). Motif analysis by MEME also indicated that the structures of the PHT1 genes were relatively conserved in different angiosperms (S4 Fig). In addition, the Ka/Ks values for all the *BnaPHT1*s were < 1.0 (S5 Fig), indicating that the paralogus PHT1 gene pairs were undergoing purifying selection during evolution.

### Multiple transcriptional regulation of the *BnaPHT1* transporters

PHT1 proteins are the best studied plant PTs [6]. These proteins may be involved in Pi uptake from the soil as well as Pi allocation from the roots to the shoots or other processes that have not yet been characterized [7, 9–11]. In this study, we identified 49 PHT1 genes in *B*. *napus* (S2 Table). Based on both RNA-seq and qRT-PCT data, we found that identified several were strongly induced by P stress (Fig 4, S6 Fig). *BnaPT11*, which is a homolog of *AtPHT1;4*, is reported to be involved in Pi uptake and seed germination [28, 29]. Among 19 homologs of *AtPHT1;1* in *B*. *napus*, *BnaPT3*, *BnaPT21*, *BnaPT25* and *BnaPT43* have the highest abundance in the roots under both P-sufficient and P-stress conditions (Figs 1 and 4, S6 Fig), indicating their vital roles in P uptake, as suggested by their homologs in *Arabidopsis* [9].

It is well documented that the expression of ion transporters might be involved in a process that influences mineral nutrient homeostasis because of the cross-talk among ion signals in response to different nutrient stresses [21, 44–46]. For instance, the expression of some Pi, K and Fe transporters is upregulated by deficiencies in these three nutrients [44]. The expression of the 14 *GmPTs* in soybean differed not only in response to P availability but also in response to other nutrient stresses, including N, K and Fe deficiencies [21]. However, the transcriptional regulation of the PHT1 genes in response to P stress and other mineral nutrient stresses in *B*. *napus* has not yet been elucidated. In this study, we investigated the expression profiles of 49 PHT1 genes in *B*. *napus* leaves and roots under P, K, N, S and Fe deprivations (Figs 4–6). Generally, the majority of *BnaPHT1*s were expressed in the roots (S6 Fig), which is in line with their major role in Pi uptake from the soil. However, some genes, such as *BnaPT31* and *BnaPT35*, also had high expression levels in the leaves (S6 Fig). It is reasonable to consider that PHT1 genes may be involved in other processes such as P redistribution and mobilization from source to sink organs [47]. Like many Pi starvation-induced genes, most PTs are transcriptionally induced by Pi deprivation [5, 9, 10]. Gene coexpression analysis based on the RNA-seq data showed that *BnaPHT1*s can cooperate with each other and other genes to regulate P homeostasis in *B*. *napus* (Fig 7). In addition to P deprivation, N, K, S and Fe starvations can also regulate the transcriptional levels of *BnaPHT1*s. Our results showed that 19, 22, 25 and 29 of the 49 *BnaPHT1*s were differentially expressed under N, K, S and Fe stresses, respectively. Forty-two *BnaPHT1* members were affected by at least two types of mineral nutrient deficiencies in the leaves and/or roots. Some PHT1 genes, such as *BnaPT48*, can simultaneously respond to five nutrient stress conditions (Fig 4, S6 Fig). These results suggest that in addition to functioning in Pi uptake and translocation, *BnaPHT1*s might be involved in cross-talk for sensing the external status of N, K, S and Fe and the synergistic regulation of N, K, S and Fe homeostasis in *B*. *napus*. However, the underlying mechanisms involved in these processes need to be further elucidated.

Soil salinity and drought are two major problems worldwide for agriculture that exert their malicious effects mainly by disrupting the ionic and osmotic equilibria of cells. To cope with these stresses, and to guarantee success in the adaptation to and survival under limiting growth conditions, plants have developed elaborate mechanisms to perceive external signals and manifest adaptive responses with proper physiological changes [48, 49]. The proper regulation of stress-responsive genes is one of the strategies for stress perception and plant responses to stress conditions. For example, 56 genes in *B*. *rapa* encode putative transcription factors whose expression is altered under cold, salt, and drought stresses [50]. Plant PTs were also reported to respond to drought and salt stresses [51, 52]. By expression analysis, we found that 24 and 19 genes were influenced by salt stress in the leaves and roots, respectively, and most of them were upregulated (Fig 5). Under drought stress conditions, 18 genes were upregulated in the leaves, and 11 genes were downregulated in the roots (Fig 5). The expression of some genes was enhanced both by salt and drought stresses (S6 Fig). These results suggest that of cross-talk occurs among the P-starvation response and salt and drought stress responses, and Pi uptake in plants likely changes in association with the altered expression of the PHT1 genes under drought and salt stresses.

It is likely that the dynamic expression profiles of PHT1 proteins require regulatory elements located in their promoters. *In silico* analyses of the CAEs in the promoters of stress-responsive genes have helped to understand the molecular and regulatory mechanisms of cross-talk among several stress signaling pathways [15]. Thus, we further identified CAEs involved in P homeostasis (P1BS and W-box elements), the salt stress response (GT-1 elements and DREs), and the drought stress response (MYCR and MBS elements) in the 2.0-kb promoter regions of 49 *BnaPHT1*s. In total, 406 CAEs in the promoters of 49 *BnaPHT1*s were detected (Fig 8, S5 Table). *In silico* analysis showed that the expression of PTs could be regulated by P deprivation as well as by salt and drought stresses. This finding was further confirmed by expression data from the qRT-PCR analysis (Figs 4–6, S6 Fig). A yeast one-hybrid assay indicated that *PHR1*, the key regulator in P homeostasis, could bind to the P1BS element in the promoter of *BnPHT1;4* (*BnaPT11* in this study) to regulate its transcription [28]. However, further analysis showed that the expression of *BnaPHT1*s was not strongly correlated with the number of CAEs in the promoter regions. This result may have occurred because TFs can regulate genes by binding to CAEs depending on other factors, such as temporo-spatial expression or individual motif activity, which vary greatly among the elements of different coregulated genes [53].

### Interactions between phytohormones and Pi signaling in regulating Pi uptake in *B*. *napus*

Plant hormones indirectly participate in the regulation of plant growth and development as signaling factors. There is extensive evidence supporting the involvement of phytohormones in nutrient signaling [15]. Auxin and CTK have been implicated in Pi signaling and in the regulation of some components of Pi starvation response pathways [15, 18, 42]. CTK can negatively regulate a number of Pi starvation-induced genes. For example, CTK suppressed the expression of a reporter gene driven by the *AtPT1* promoter [54]. Ausin signaling has been suggested to be associated closely with the modification of root architecture caused by Pi deprivation [20]. Moreover, it has been reported that the expression levels of *OsPT8* in the roots, root-shoot junctions and leaves of rice were induced by IAA, revealing a novel biological function of *OsPT8* in the cross-talk between Pi and auxin signaling [13]. Using *in silico* analysis, Baek et al. (2017) reported that a number of CAEs related to the response to auxin, GA, ethylene, JA, SA and ABA ocurred in the PHT1 promoter regions in *Arabidopsis*, indicating the possible interactions between these phytohormones and Pi signaling [15]. In the present study, we identified three CAEs that were related to auxin in the *B*. *napus* PHT1 promoters. In total, 44 CAEs were found in 49 *BnaPHT1*s (S5 Table). To further examine the response of *BnaPHT1*s to auxin and CTK, the transcript abundance of 49 *BnaPHT1*s was determined by qRT-PCR under exogenous IAA and CTK treatments. Our results showed that among the 49 *BnaPHT1*s, 36 were affected by IAA, and 27 were influenced by CTK (Fig 6), indicating the interactions between phytohormones and Pi signaling in the regulation of Pi uptake in *B*. *napu*s. Moreover, the expression of 49 *BnaPHT1*s was enhanced, or repressed, or unaffected by different hormones, indicative of functional differentiation for different *B*. *napus* PHT1 family members in the signaling pathways of hormones and P homeostasis. Our results also point to the complex nature of hormone interactions during Pi starvation in the regulation of plant Pi uptake, which needs to be further studied.

## Supporting information

S1 Fig The expression levels of marker genes in rapeseed seedlings under different stress conditions. Nine genes, *BnaNRT2*.*5* (BnaA08g24500D), *BnaSPX3* (BnaC03g25110D), *BnaSultr1;1* (BnaC07g18000D), *BnaNAC2* (BnaC06g30680D), *BnaIAA9* (BnaC03g39170D), *BnaARR5* (BnaC01g42890D), *BnaDi19* (BnaC07g28390D), *BnaHAK5* (BnaC06g15440D), *BnaFRD1* (BnaA10g00390D) were selected as marker genes for different treatments. Values are means ± SD of three biological replicates. Asterisks indicate significant difference at * *P* < 0.05, ** *P* < 0.01 by Student’s *t* test, respectively.
(TIF)

S2 Fig Chromosomal locations of *BnaPHT1*s, *BrPHT1*s and *BoPHT1*s. The 46 *BnaPHT1*s, 24 *BrPHT1*s and 23 *BoPHT1*s for which exact chromosomal information was available in the database were mapped to the chromosomes. A, gene location in the chromosomes of *Brassica napus* A subgenome. B, gene location in the chromosomes of *B*. *rapa* genome. C, gene location in the chromosomes of *B*. *napus* C subgenome. D, gene location in the chromosomes of *B*. *oleracea* genome. The arrows indicate the direction of transcription. The diagram was drawn using the MapInspect software.
(TIF)

S3 Fig Phylogenetic relationship and gene structure of the PHT1 family genes in *Arabidopsis*, *Brassica oleracea*, *B*. *rapa* and *B*. *napus*. The exon-intron structures of PTs were determined by the alignments of coding sequences with corresponding genomic sequences. The diagram was obtained using GSDS web server (http://gsds.cbi.pku.edu.cn/). Colored boxes indicate the exons of PTs, while gray lines represent the introns. The amino acid sequences of PHT1s from four species were aligned using ClustalW, and the phylogenetic tree was constructed using MEGA 5.2 with the neighbor-joining method (1000 bootstrap replicates).
(TIF)

S4 Fig **Distribution of conserved motifs and WebLogo plots of the consensus motifs in *BnaPHT1* family (A), *BrPHT1* family (B), *BoPHT1* family (C) and *AtPHT1* family (D).** Conserved motifs of the PHT1 family members for four species were analyzed by MEME Web service (http://alternate.meme-suite.org/) using the protein sequences. Fifteen conserved motifs (E) were identified, and different motifs were distinguished by colored boxes. Boxed sequence (GGDYPLSATIMSE) in motif 3 is the conserved PHT1 signature (E).
(TIF)

S5 Fig The synonymous nucleotide substitution rates (Ks) and non-synonymous nucleotide substitution rates (Ka) of the PHT1 family proteins in *Brassica napus*. The values of Ks, Ka and Ka/Ks are shown. The X axis indicates different PHT1 family proteins in *B*. *napus*, and the Y axis is denoted by the values of Ka, Ks and Ka/Ks.
(TIF)

S6 Fig Expression analysis of the PHT1 family genes in *Brassica napus*. Seedlings of 14 days old were exposed to various growth environments for six days except drought stress (three days). The fully expanded leaf and roots were sampled separately for RNA extraction. CK, full strength Hoagland’s solution. -P, no phosphorus; -N, no nitrogen. -K, no potassium. -S, no sulfur. -Fe, no iron. NaCl, salt stress. PEG, drought stress. IAA, auxin. CTK, cytokinin. Data are means ± SD with three biological replicates. * and ** indicates significant difference at *P* < 0.05 and *P* < 0.01 by Duncan’s test, respectively.
(TIF)

S1 Table Primers used for quantitative real-time PCR used in this study. (DOCX)

S2 Table Characteristics of the PHT1 family members in *Brassica napus* and subcellular localization prediction. (DOCX)

S3 Table Copy number variations (CNVs) of the PHT1 family genes in Viridiplantae. (DOCX)

S4 Table Tandemly duplicated *BnaPHT1* genes in *Brassica napus*. (DOCX)

S5 Table Number of cis-elements in the 2.0-kb promoter regions of the PHT1 family genes in *Brassica napus*. (DOCX)
