# Supplementary figures and images for: Molecular identification of the phosphate transporter family 1 (PHT1) genes and their expression profiles in response to phosphorus deprivation and other abiotic stresses in Brassica napus
Source: PLoS One. 2019 Jul 25;14(7):e0220374. doi: 10.1371/journal.pone.0220374 (PMC6657917; doi:10.1371/journal.pone.0220374)

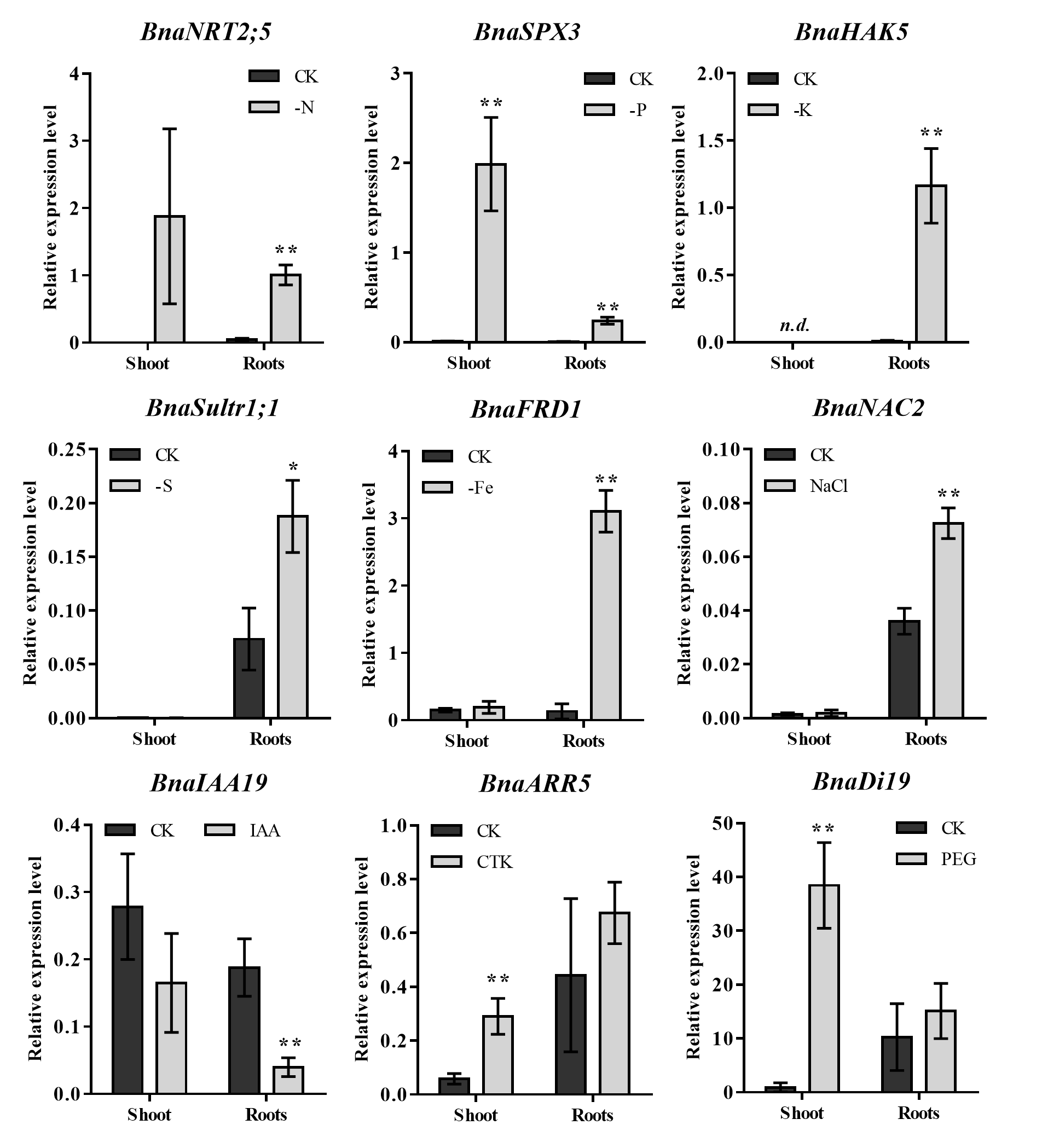

Supplement: S1 Fig — Nine genes, BnaNRT2.5 (BnaA08g24500D), BnaSPX3 (BnaC03g25110D), BnaSultr1;1 (BnaC07g18000D), BnaNAC2 (BnaC06g30680D), BnaIAA9 (BnaC03g39170D), BnaARR5 (BnaC01g42890D), BnaDi19 (BnaC07g28390D), BnaHAK5 (BnaC06g15440D), BnaFRD1 (BnaA10g00390D) were selected as marker genes for different treatments. Values are means ± SD of three biological replicates. Asterisks indicate significant difference at * P < 0.05, ** P < 0.01 by Student’s t test, respectively. (TIF) [file pone.0220374.s001.tif]

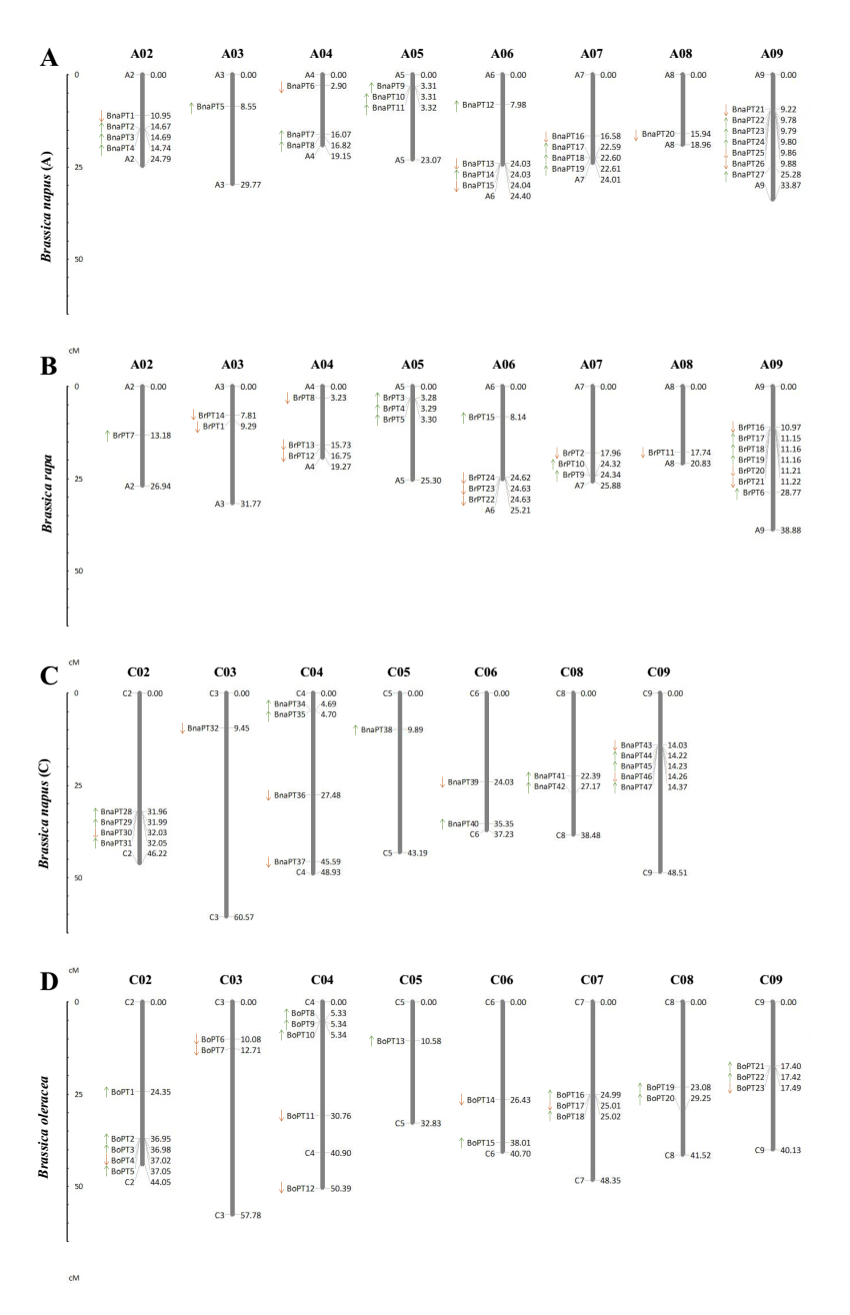

Supplement: S2 Fig — The 46 BnaPHT1s, 24 BrPHT1s and 23 BoPHT1s for which exact chromosomal information was available in the database were mapped to the chromosomes. A, gene location in the chromosomes of Brassica napus A subgenome. B, gene location in the chromosomes of B. rapa genome. C, gene location in the chromosomes of B. napus C subgenome. D, gene location in the chromosomes of B. oleracea genome. The arrows indicate the direction of transcription. The diagram was drawn using the MapInspect software. (TIF) [file pone.0220374.s002.tif]

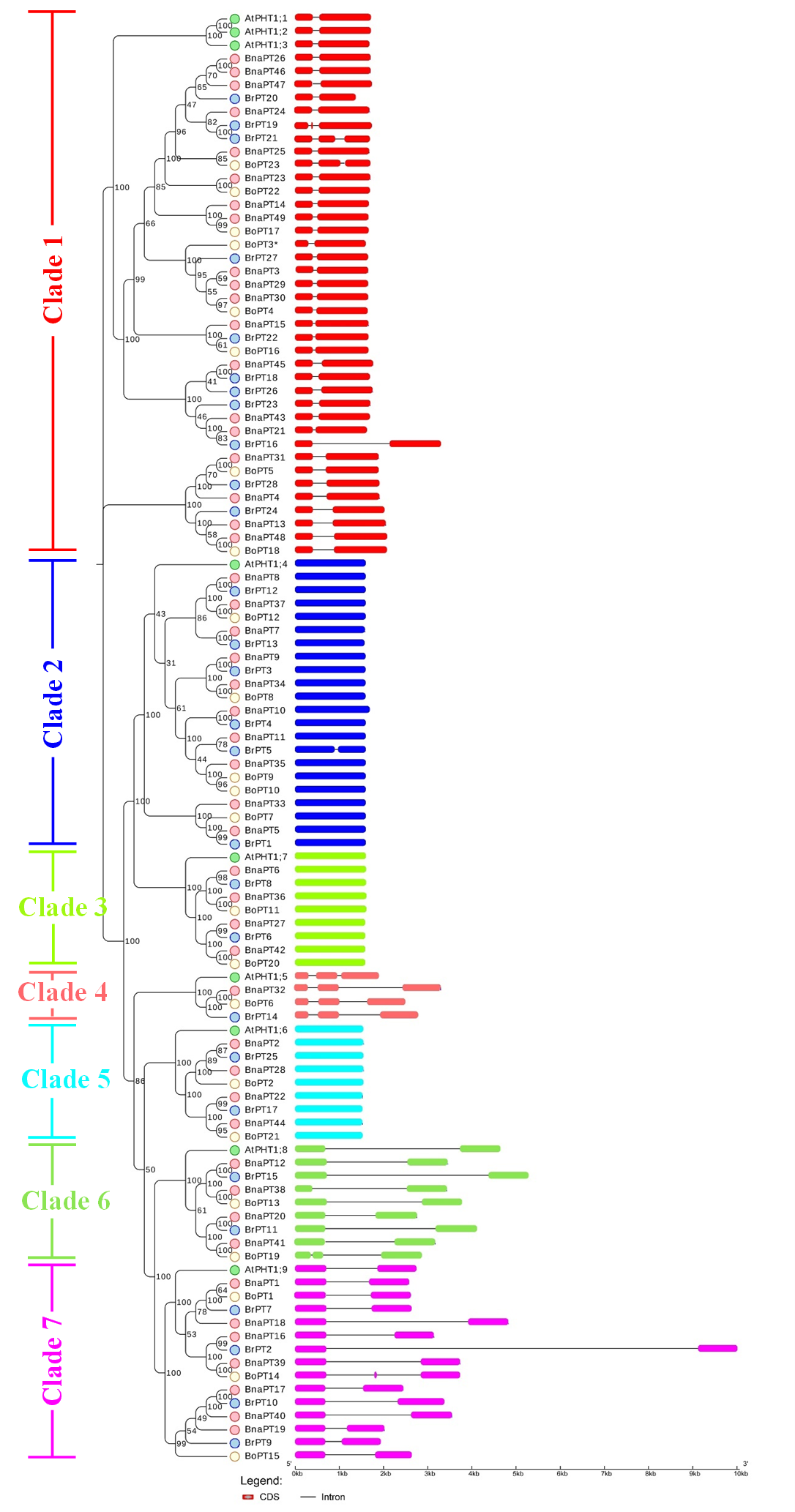

Supplement: S3 Fig — The exon-intron structures of PTs were determined by the alignments of coding sequences with corresponding genomic sequences. The diagram was obtained using GSDS web server (http://gsds.cbi.pku.edu.cn/). Colored boxes indicate the exons of PTs, while gray lines represent the introns. The amino acid sequences of PHT1s from four species were aligned using ClustalW, and the phylogenetic tree was constructed using MEGA 5.2 with the neighbor-joining method (1000 bootstrap replicates). (TIF) [file pone.0220374.s003.tif]

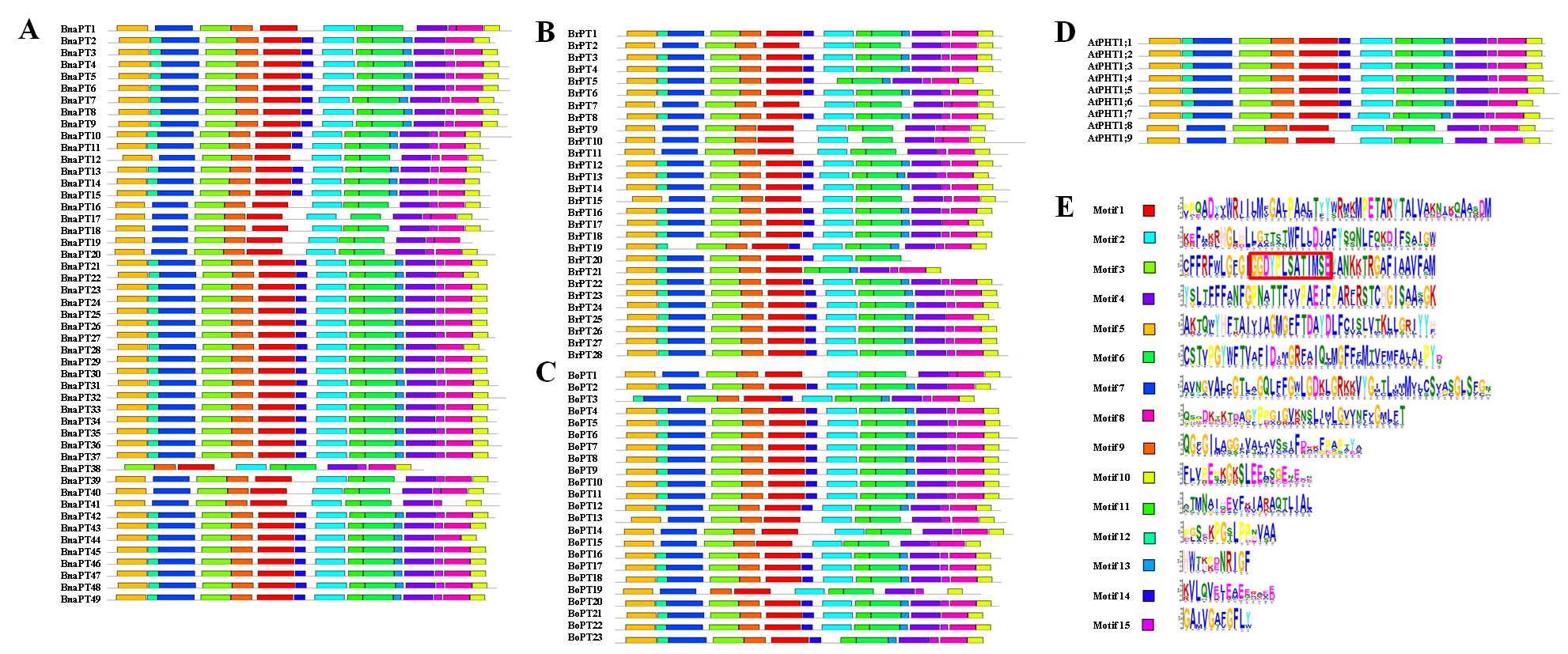

Supplement: S4 Fig — Distribution of conserved motifs and WebLogo plots of the consensus motifs in BnaPHT1 family (A), BrPHT1 family (B), BoPHT1 family (C) and AtPHT1 family (D). Conserved motifs of the PHT1 family members for four species were analyzed by MEME Web service (http://alternate.meme-suite.org/) using the protein sequences. Fifteen conserved motifs (E) were identified, and different motifs were distinguished by colored boxes. Boxed sequence (GGDYPLSATIMSE) in motif 3 is the conserved PHT1 signature (E). (TIF) [file pone.0220374.s004.tif]

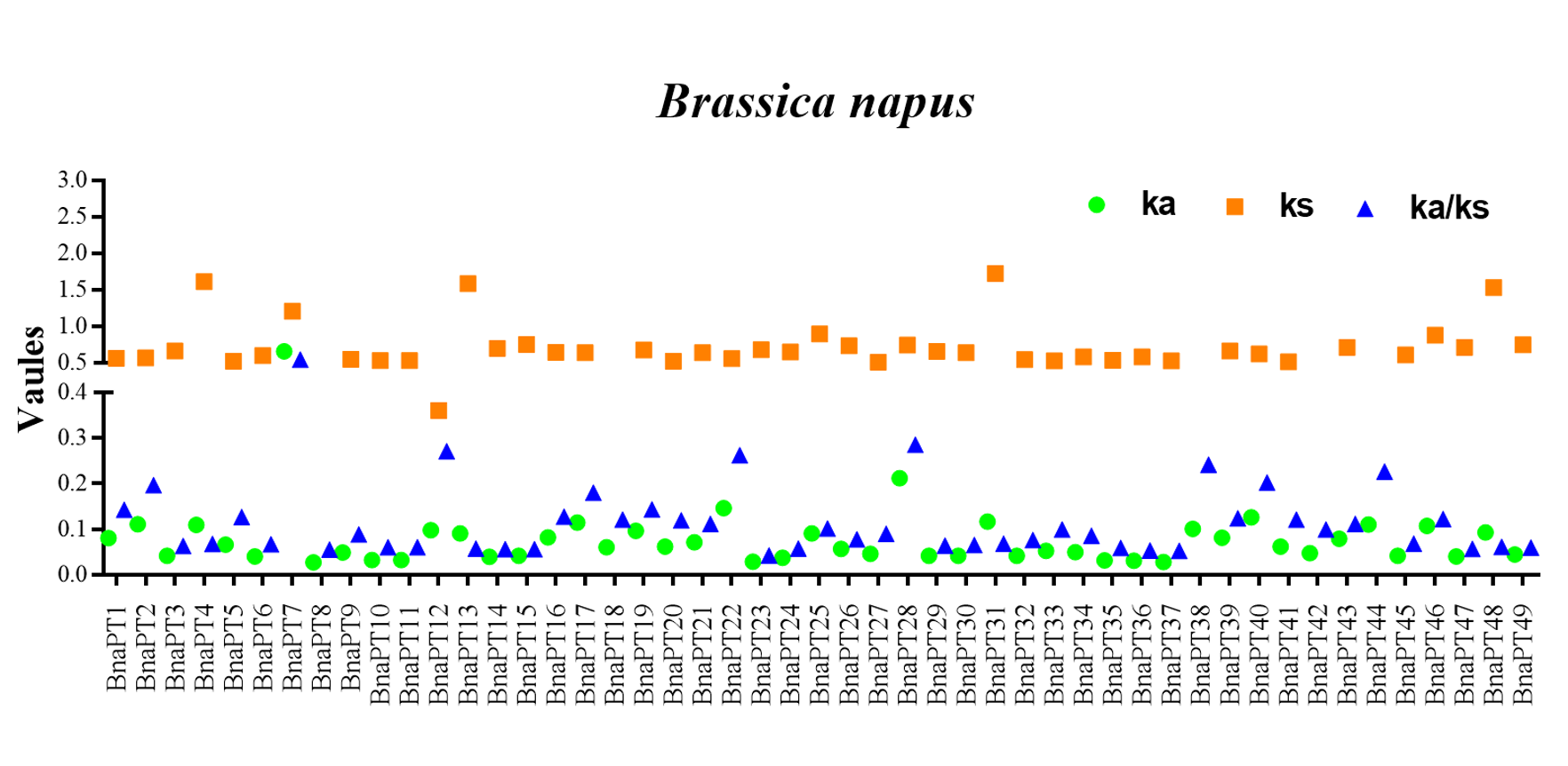

Supplement: S5 Fig — The values of Ks, Ka and Ka/Ks are shown. The X axis indicates different PHT1 family proteins in B. napus, and the Y axis is denoted by the values of Ka, Ks and Ka/Ks. (TIF) [file pone.0220374.s005.tif]

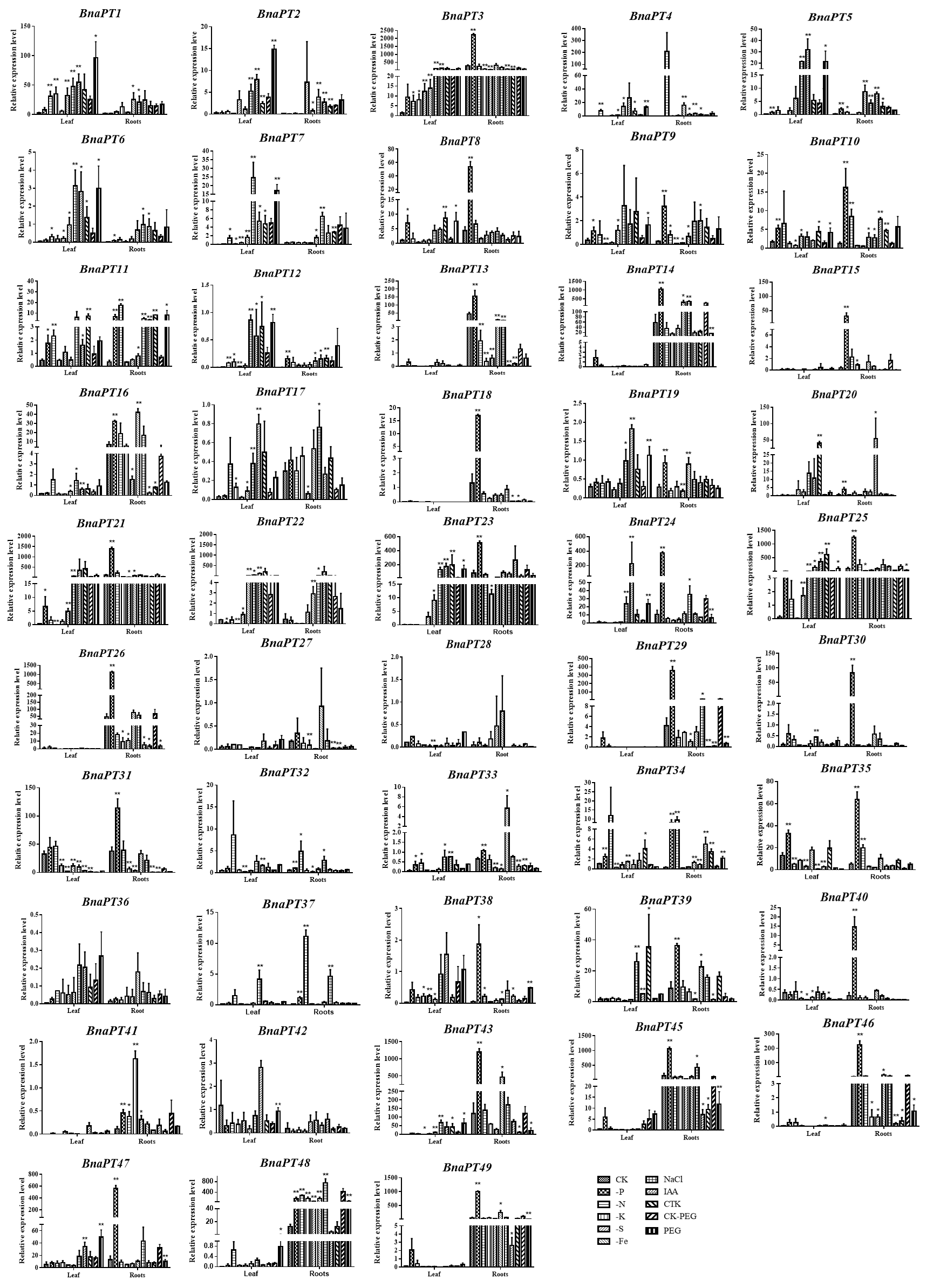

Supplement: S6 Fig — Seedlings of 14 days old were exposed to various growth environments for six days except drought stress (three days). The fully expanded leaf and roots were sampled separately for RNA extraction. CK, full strength Hoagland’s solution. -P, no phosphorus; -N, no nitrogen. -K, no potassium. -S, no sulfur. -Fe, no iron. NaCl, salt stress. PEG, drought stress. IAA, auxin. CTK, cytokinin. Data are means ± SD with three biological replicates. * and ** indicates significant difference at P < 0.05 and P < 0.01 by Duncan’s test, respectively. (TIF) [file pone.0220374.s006.tif]
