# Supplementary material for: Molecular identification of the phosphate transporter family 1 (PHT1) genes and their expression profiles in response to phosphorus deprivation and other abiotic stresses in Brassica napus
Source: PLoS One. 2019 Jul 25;14(7):e0220374. doi: 10.1371/journal.pone.0220374 (PMC6657917; doi:10.1371/journal.pone.0220374)
Supplement: S1 Table — (DOCX) [file pone.0220374.s007.docx]

**S1 Table. Primers used for quantitative real-time PCR used in this study.**

| **Gene name** | **Forward primer** | **Reverse primer** |
| --- | --- | --- |
| *BnaPT1* | TACCCGAAGAAGATTCGTCATCG | GATTTGAGCGAGGAGGAGGTTG |
| *BnaPT2* | TAGCAGCAGGAGGTGTTTCG | AGCGGTTTCAGGCATCTTCA |
| *BnaPT3* | GGGAGCTTTTGGATTCTTATACG | GCTCTTCAAGGGACTTACCCTTG |
| *BnaPT4* | GCGAACAAGAAGACTCGTGGTG | ATGGGCGACGGGAATTGGT |
| *BnaPT5* | ATTGCCTTGTGTAGCACGATG | CGGTTCTCCTTGTGAGTCCAGT |
| *BnaPT6* | CTAAAAGAGAGCAATGGCAGGAG | CGTGAGTGTGCCAACAAAT |
| *BnaPT7* | TTCTTGTACTTGTCCCAAAGTCCT | CGGACATTTCCTCAAGCGACT |
| *BnaPT8* | CGGGAGAAAGAAAGTCTATGGTAT | ATCGTTGCGGACAAAGGGTAG |
| *BnaPT9* | GCAATTCATGAAACGCCATGGACTTCAC | CTGCCGGCACGACGAATGTTGTAGC |
| *BnaPT10* | TTTGCTTTGGCTATTCCGTAT | ATCTCCGCCGGTACAACG |
| *BnaPT11* | ATCAGGAAAGTTAGGTGCC | AAGAGGATGCCCAAGAAGT |
| *BnaPT12* | CTTTGGCGGTGGTTCGAAGGCT | GGCATCAACATTCGCCAGTATAAGGTC |
| *BnaPT13* | TTCAAGATCCAAAGCTTAACTAT | CACCCAATGGCAGAAAATAT |
| *BnaPT14* | GCTAGGGCACAAACACTCATT | AGTACATAACAACGAAGCCGATA |
| *BnaPT15* | ATCTTGCGAAAACCCAACTTTAC | GTTGACCGGCAAGGGTCC |
| *BnaPT16* | CCTAGTCGCTGGGGTTCCGTACAG | CTCCGGATATCCCATGACAAGTTGAC |
| *BnaPT17* | CACAACTACCATCGTCTTCCTCTTC | CATTGTGAGGTTTGTTGGATACATTGAGG |
| *BnaPT18* | ATAGCCGAAGATACATCATCAGAGC | GTTACTTGTGTAGAAGACCACGTCG |
| *BnaPT19* | TCAACTTGCCACGGGATTTC | CGTCACAAACATTCCAGCGATA |
| *BnaPT20* | GTCGCAGGGATACCGTACACG | CTAAACCTAGCCGGGAAAAGCTCT |
| *BnaPT21* | ATCAGCCGCAACAGGTAAGG | AGGGACAAGGAAGGTGAAGAG |
| *BnaPT22* | CAGGCAGATTACGTGTGGAGG | CCTTAGACATATCTGTAGCCGCTTT |
| *BnaPT23* | CGTATCGGCTTCGTTGTTAT | CATCCGTCTTGGTCTTGTCTT |
| *BnaPT24* | CCTGAAACTGCTCGTTACACC | CCTCTGCTCTTTCCTCCATCTC |
| *BnaPT25* | GCGATTGCCTTCCCTTACGA | CTTGCCTGTTGCGGCTGATA |
| *BnaPT26* | TGGATGGCTGGGTGACAAGCTCGGAAG | GAAACCTCCGGCTAAGATACCAACACC |
| *BnaPT27* | ATGACTGTCTTTATGTTCGCTCTA | TGTCAGGACTCTGTGCCAAGTA |
| *BnaPT28* | GACAAGTGGACCTAAGTCTAGCC | GACCTTACCTCATAACCGGATT |
| *BnaPT29* | GGGAGCTTTTGGATTCTTATACG | CTCTTCAAGGGACTTGCCC |
| *BnaPT30* | GATGCTTATGATCTGTTTTGCGTG | GCAAGGGTCCCACAGAGAG |
| *BnaPT31* | TGTTTGCTCTGTCGCCTCCG | TGCCTCCAGCCAAGATTCCA |
| *BnaPT32* | GCAGAGTACTGATCCGAAGAAGAC | CTGCTCCTCATCCTCTCTTG |
| *BnaPT33* | CATTCTTTTTTGCCAACTTTGGG | CTTCAGGCACCAAGAAAGTGAAT |
| *BnaPT34* | ACAATTCATGAAACGCCATGGACTTC | GTGTACGGAATAGCCAACGCAAAC |
| *BnaPT35* | GATAGAGGCTGAACAGGAGAAA | AGAAAGCGATGTCGAGAAGGA |
| *BnaPT36* | GGACCTAATGCCACAACCTTTGTG | GAGTTCTTCACACCAATCCCG |
| *BnaPT37* | TCGGGAGGAAGAAAGTTTATGG | TGATCGTTGCAGACAAAGGGTAG |
| *BnaPT38* | CATCATCGCCGCTTGCTCT | CGTAAAGAACCATAAAGCCTTTCTC |
| *BnaPT39* | GTTGTTTACCTGGTTGCTGGG | GATATCCCGTGACAAGTTGACCTA |
| *BnaPT40* | TGTATCGCTGGAGTGTTGGTG | TTGCTTGCGTTGTAATTCTCG |
| *BnaPT41* | GAGTTTGCATTGCTGGAATATTCG | CAACAATATGTGGCTCATCTTCA |
| *BnaPT42* | GGCTGCTTTGTCTCTTGTGTTCTTGG | GCACCGTGAATCATCTTCTCTTGTGG |
| *BnaPT43* | GCCACAGGACAAGACCAAGA | TGAAGAGCATACCGACGAAGT |
| *BnaPT44* | TTTTCGCTATGCAAGGATTTG | CTTCGCACGCCAGTAGTAAGT |
| *BnaPT45* | GGTTCACGGTTGCGTTTATTG | TGGTTTGATCCAATGGTCGT |
| *BnaPT46* | ATGGCCGAACAACAACTAGGA | CTGAAGAAGCAAAGAGTGGTC |
| *BnaPT47* | GCTAGGGCACAAACACTCATTG | TGGTCGTAAGGGAAGGCAAT |
| *BnaPT48* | TATTATTGGCGAATGAAGATGC | CCTTGGAGAACAAGCCGTAG |
| *BnaPT49* | GCTAGGGCACAAACACTCATTG | AGAGTACATAACAACGAAGCCGATA |
| *BnaNRT2;5* | TTATCGCACGAGAACAAAG | TTCCACAATGGGGAGGTATG |
| *BnaSPX3* | GTGAGTTCAGCGGCTGCG | CTAACGACATCTTCCTAGAACGAC |
| *BnaHAK5* | GGGCATTGCTCTATTCTACACG | CTCCTCTTTCTCCGCCTCTTC |
| *BnaSultr1;1* | CTCTTCAAGTACACGCCAAACG | CGCAACCATCTTAGAATCCTTTC |
| *BnaFRD1* | TGGAGCTTTATCACTTACTTGCG | GTTATGCCGACGTGGAGGAC |
| *BnaNAC2* | GTCGACGAAACTTCCGAGGAC | ACAATCTTCCATGTTCATTTC |
| *BnaDi19* | TCTTCGTTCATCACGCCAATG | AAGCGACTTCTTCTCAACCTTCC |
| *BnaIAA19* | AGGTTATGATGATTTAGCCTTTGC | CACCGTATCACTCGTCTACTCCTCTA |
| *BnaARR5* | CCTGTGAAGTTGGCTGATGTG | TCAGGAAATTCTAAAGTGCGTGT |
| *EF1-α* | GCCTGGTATGGTTGTGACCT | GAAGTTAGCAGCACCCTTGG |
