# Supplementary material for: Molecular identification of the phosphate transporter family 1 (PHT1) genes and their expression profiles in response to phosphorus deprivation and other abiotic stresses in Brassica napus
Source: PLoS One. 2019 Jul 25;14(7):e0220374. doi: 10.1371/journal.pone.0220374 (PMC6657917; doi:10.1371/journal.pone.0220374)
Supplement: S2 Table — (DOCX) [file pone.0220374.s008.docx]

**S2 Table. Characteristics of the PHT1 family members in *Brassica napus* and subcellular localization prediction.**

| **Gene name** | **Gene ID** | **Gene position** | | **Gene length (bp)** | **Protein length (aa)** | **Molecular weight (kDa)** | **GRAVY** | **pI** | **Intron number** | **Subcellular localization (Wolf Psort)** |
| --- | --- | --- | --- | --- | --- | --- | --- | --- | --- | --- |
|  |  | **Start** | **End** |  |  |  |  |  |  |  |
| *BnaPT1* | BnaA02g18110D | 10952409 | 10955002 | 2594 | 539 | 59.22 | 0.36 | 8.91 | 1 | plasma membrane |
| *BnaPT2* | BnaA02g22170D, | 14668895 | 14670448 | 1554 | 517 | 56.38 | 0.473 | 8.95 | 0 | plasma membrane |
|  | BnaA02g22180D |  |  |  |  |  |  |  |  |  |
| *BnaPT3* | BnaA02g22230D | 14686594 | 14688254 | 1661 | 521 | 57.25 | 0.417 | 9.08 | 1 | plasma membrane |
| *BnaPT4* | BnaA02g22250D | 14736673 | 14738592 | 1920 | 535 | 58.66 | 0.386 | 9.23 | 1 | vacuolar membrane |
| *BnaPT5* | BnaA03g18250D | 8553172 | 8554779 | 1608 | 535 | 58.6 | 0.395 | 9.07 | 0 | plasma membrane |
| *BnaPT6* | BnaA04g04020D | 2896429 | 2898042 | 1614 | 537 | 59 | 0.304 | 8.86 | 0 | plasma membrane |
| *BnaPT7* | BnaA04g20990D, | 16065104 | 16066687 | 1584 | 527 | 58.11 | 0.39 | 7.07 | 0 | plasma membrane |
|  | BnaA04g21000D, |  |  |  |  |  |  |  |  |  |
|  | BnaA04g21010D |  |  |  |  |  |  |  |  |  |
| *BnaPT8* | BnaA04g22280D | 16822046 | 16823650 | 1605 | 534 | 58.5 | 0.363 | 8.81 | 0 | plasma membrane |
| *BnaPT9* | BnaA05g06200D | 3307014 | 3308615 | 1602 | 533 | 58.82 | 0.309 | 8.56 | 0 | plasma membrane |
| *BnaPT10* | BnaA05g06210D | 3310985 | 3312682 | 1698 | 565 | 62.09 | 0.433 | 8.82 | 0 | plasma membrane/ vacuolar membrane |
| *BnaPT11* | BnaA05g06230D | 3318785 | 3320389 | 1605 | 534 | 58.52 | 0.362 | 8.81 | 0 | plasma membrane |
| *BnaPT12* | BnaA06g14710D, | 7977184 | 7980654 | 3471 | 544 | 60 | 0.306 | 8.55 | 1 | plasma membrane |
|  | BnaA06g14720D |  |  |  |  |  |  |  |  |  |
| *BnaPT13* | BnaA06g36740D | 24028673 | 24030733 | 2061 | 535 | 58.31 | 0.394 | 8.88 | 1 | plasma membrane |
| *BnaPT14* | BnaA06g36750D | 24032309 | 24033985 | 1677 | 521 | 57.25 | 0.417 | 9.15 | 1 | plasma membrane |
| *BnaPT15* | BnaA06g36760D | 24036240 | 24037909 | 1670 | 535 | 58.9 | 0.402 | 8.93 | 1 | plasma membrane |
| *BnaPT16* | BnaA07g21370D | 16579573 | 16582729 | 3157 | 534 | 59.03 | 0.421 | 6.62 | 1 | plasma membrane |
| *BnaPT17* | BnaA07g32730D | 22592953 | 22595415 | 2463 | 533 | 58.44 | 0.398 | 8.82 | 1 | plasma membrane |
| *BnaPT18* | BnaA07g32740D | 22596590 | 22601439 | 4850 | 540 | 59.46 | 0.357 | 8.71 | 1 | plasma membrane |
| *BnaPT19* | BnaA07g32750D | 22608377 | 22610407 | 2031 | 511 | 56.68 | 0.308 | 9.09 | 1 | plasma membrane |
| *BnaPT20* | BnaA08g21590D | 15942977 | 15945749 | 2773 | 542 | 59.78 | 0.326 | 6.27 | 1 | plasma membrane |
| *BnaPT21* | BnaA09g15770D | 9219242 | 9221016 | 1775 | 521 | 57.15 | 0.411 | 9.1 | 1 | plasma membrane |
| *BnaPT22* | BnaA09g16420D, | 9775617 | 9777149 | 1533 | 510 | 55.53 | 0.457 | 8.8 | 0 | plasma membrane |
|  | BnaA09g16430D |  |  |  |  |  |  |  |  |  |
| *BnaPT23* | BnaA09g16440D | 9793366 | 9795077 | 1712 | 521 | 57.29 | 0.398 | 9.11 | 1 | plasma membrane |
| *BnaPT24* | BnaA09g16450D, | 9798141 | 9799850 | 1710 | 521 | 57.24 | 0.416 | 9.14 | 1 | plasma membrane |
|  | BnaA09g16460D |  |  |  |  |  |  |  |  |  |
| *BnaPT25* | BnaA09g16490D | 9858907 | 9860610 | 1704 | 521 | 57.25 | 0.416 | 9.14 | 1 | plasma membrane |
| *BnaPT26* | BnaA09g16500D | 9884098 | 9885814 | 1717 | 521 | 57.25 | 0.416 | 9.14 | 1 | plasma membrane |
| *BnaPT27* | BnaA09g34510D | 25276148 | 25277743 | 1596 | 531 | 58.24 | 0.342 | 7.65 | 0 | plasma membrane |
| *BnaPT28* | BnaC02g30190D, | 31958928 | 31960481 | 1554 | 517 | 56.52 | 0.478 | 9.03 | 0 | plasma membrane |
|  | BnaC02g30200D |  |  |  |  |  |  |  |  |  |
| *BnaPT29* | BnaC02g30230D | 31993562 | 31995222 | 1661 | 521 | 57.25 | 0.417 | 9.08 | 1 | plasma membrane |
| *BnaPT30* | BnaC02g30240D | 32028564 | 32030224 | 1661 | 521 | 57.25 | 0.417 | 9.08 | 1 | plasma membrane |
| *BnaPT31* | BnaC02g30270D | 32047332 | 32049233 | 1902 | 535 | 58.59 | 0.39 | 9.33 | 1 | vacuolar membrane |
| *BnaPT32* | BnaC03g18410D | 9446975 | 9450304 | 3330 | 546 | 59.32 | 0.316 | 8.55 | 2 | plasma membrane |
| *BnaPT33* | BnaC03g72240D | 596327 | 597931 | 1605 | 534 | 58.4 | 0.398 | 8.7 | 0 | plasma membrane |
| *BnaPT34* | BnaC04g06470D | 4691008 | 4692609 | 1602 | 533 | 58.66 | 0.335 | 8.56 | 0 | plasma membrane |
| *BnaPT35* | BnaC04g06480D | 4695993 | 4697597 | 1605 | 534 | 58.54 | 0.362 | 8.81 | 0 | plasma membrane |
| *BnaPT36* | BnaC04g26100D | 27475008 | 27476630 | 1623 | 540 | 59.24 | 0.314 | 8.86 | 0 | plasma membrane |
| *BnaPT37* | BnaC04g46050D | 45585366 | 45586970 | 1605 | 534 | 58.47 | 0.364 | 8.8 | 0 | plasma membrane |
| *BnaPT38* | BnaC05g16120D | 9894902 | 9898355 | 3454 | 433 | 47.72 | 0.303 | 6.09 | 1 | plasma membrane |
| *BnaPT39* | BnaC06g21850D | 24026340 | 24030093 | 3754 | 534 | 59.01 | 0.416 | 6.62 | 1 | plasma membrane |
| *BnaPT40* | BnaC06g37180D | 35348219 | 35351786 | 3568 | 538 | 59.08 | 0.308 | 8.61 | 1 | plasma membrane |
| *BnaPT41* | BnaC08g19520D | 22393763 | 22396949 | 3187 | 537 | 59.16 | 0.306 | 5.77 | 1 | plasma membrane |
| *BnaPT42* | BnaC08g25460D | 27169466 | 27171061 | 1596 | 531 | 58.26 | 0.346 | 7.65 | 0 | plasma membrane |
| *BnaPT43* | BnaC09g17240D | 14027831 | 14029594 | 1764 | 521 | 57.14 | 0.391 | 9.2 | 1 | plasma membrane |
| *BnaPT44* | BnaC09g17490D, | 14219330 | 14220859 | 1530 | 509 | 55.45 | 0.461 | 8.63 | 0 | plasma membrane |
|  | BnaC09g17500D |  |  |  |  |  |  |  |  |  |
| *BnaPT45* | BnaC09g17510D | 14225602 | 14227313 | 1712 | 521 | 57.38 | 0.382 | 9.07 | 1 | vacuolar membrane |
| *BnaPT46* | BnaC09g17550D | 14263767 | 14265483 | 1717 | 521 | 57.22 | 0.421 | 9.14 | 1 | plasma membrane |
| *BnaPT47* | BnaC09g17610D | 14370796 | 14372541 | 1746 | 521 | 57.22 | 0.411 | 9.14 | 1 | plasma membrane |
| *BnaPT48* | BnaCnng37050D | 35394874 | 35396967 | 2094 | 535 | 58.36 | 0.378 | 8.99 | 1 | plasma membrane |
| *BnaPT49* | BnaCnng37060D | 35411092 | 35412761 | 1670 | 520 | 57.12 | 0.424 | 9.15 | 1 | plasma membrane |
