# Supplementary material for: Molecular identification of the phosphate transporter family 1 (PHT1) genes and their expression profiles in response to phosphorus deprivation and other abiotic stresses in Brassica napus
Source: PLoS One. 2019 Jul 25;14(7):e0220374. doi: 10.1371/journal.pone.0220374 (PMC6657917; doi:10.1371/journal.pone.0220374)
Supplement: S3 Table — (DOCX) [file pone.0220374.s009.docx]

**S3 Table. Copy number variations (CNVs) of the PHT1 family genes in Viridiplantae.**

| **Species name** | **Copy number** | **Reference** | **Species name** | **Copy number** | **Reference** |
| --- | --- | --- | --- | --- | --- |
| *Arabidopsis thaliana* | 9 | Planta. 2002, 216: 23-37 | *Medicago truncatula* | 5 | Journal of biological chemistry. 2008, 283: 24673-24681 |
| *Astragalus sinicus* | 6 | New phytologist. 2013, 198: 836-852 | *Nicotiana tabacum* | 5 | New phytologist. 2007, 173 (4): 817-831 |
| *Brachypodium* | 12 | Molecular plant breeding 2011, 9: 482-490 | *Oryza sativa* | 13 | Science. 2002, 296: 92–100 |
| *Brassica napus* | 49 | BLAST search in genome database | *Populus trichocarpa* | 11 | Plant physiology. 2011, 156 (4): 2141-2154 |
|  |  |  |  | 14 | Frontiers in plant science. 2016, 7: 1398 |
| *Brassica oleracea* | 23 | BLAST search in genome database | *Salvia miltiorrhiza* | 13 | Chinese traditional and herbal drugs. 2018, 1: 194-202 |
| *Brassica rapa* | 28 | BLAST search in genome database | *Setaria italica* | 12 | PLoS ONE. 2014, 24, 9 (9): e108459 |
| *Capsicum frutescens* | 5 | New phytologist. 2007, 173 (4): 817-831 | *Solanum lycopersicum* | 8 | BMC Plant biology. 2014, 14: 61 |
| *Citrus sinensis* | 7 | Molecular plant breeding. 2017, 15 (12): 4854-4860 | *Solanum melongena* | 5 | New phytologist. 2007, 173 (4): 817-831 |
| *Glycine max* | 14 | PLoS ONE. 2012, 7 (10): e47726 | *Solanum tuberosum* | 8 | Journal of biotechnology. 2017, 264: 17-28 |
| *Gossypium hirsutum* | 17 | Cotton science. 2017, 29: 59-69 | *Sorghum bicolor* | 11 | New phytologist. 2015, 205: 1632-1645 |
| *Linum usitatissimum* | 9 | New phytologist. 2015, 205: 1632-1645 | *Triticum aestivum* | 21 | Frontiers in plant science. 2017, 8: 543 |
|  |  |  |  |  | Plant Biology. 2018, 20: 374-389 |
| *Malus domestica* | 14 | Frontiers in plant science. 2017, 8: 426 | *Zea mays* | 13 | International journal of molecular sciences. 2016, 17: 930 |
