# Supplementary material for: Molecular identification of the phosphate transporter family 1 (PHT1) genes and their expression profiles in response to phosphorus deprivation and other abiotic stresses in Brassica napus
Source: PLoS One. 2019 Jul 25;14(7):e0220374. doi: 10.1371/journal.pone.0220374 (PMC6657917; doi:10.1371/journal.pone.0220374)
Supplement: S4 Table — (DOCX) [file pone.0220374.s010.docx]

**S4 Table. Tandemly duplicated *BnaPHT1* genes in *Brassica napus.***

| Gene name | Chromosome | Gene position | | Cluster |
| --- | --- | --- | --- | --- |
|  |  | Start | End |  |
| *BnaPT9* | A05 | 3307014 | 3308615 | Cluster_1 |
| *BnaPT10* | A05 | 3310985 | 3312682 |  |
| *BnaPT11* | A05 | 3318785 | 3320389 |  |
| *BnaPT14* | A06 | 24032309 | 24033985 | Cluster_2 |
| *BnaPT15* | A06 | 24036240 | 24037909 |  |
| *BnaPT17* | A07 | 22592953 | 22595415 | Cluster_3 |
| *BnaPT19* | A07 | 22608377 | 22610407 |  |
| *BnaPT23* | A09 | 9793366 | 9795077 | Cluster_4 |
| *BnaPT24* | A09 | 9798141 | 9799850 |  |
| *BnaPT25* | A09 | 9858907 | 9860610 |  |
| *BnaPT26* | A09 | 9884098 | 9885814 |  |
| *BnaPT29* | C02 | 31993562 | 31995222 | Cluster_5 |
| *BnaPT30* | C02 | 32028564 | 32030224 |  |
| *BnaPT34* | C04 | 4691008 | 4692609 | Cluster_6 |
| *BnaPT35* | C04 | 4695993 | 4697597 |  |
| *BnaPT45* | C09 | 14225602 | 14227313 | Cluster_7 |
| *BnaPT46* | C09 | 14263767 | 14265483 |  |
