# Supplementary material for: Molecular identification of the phosphate transporter family 1 (PHT1) genes and their expression profiles in response to phosphorus deprivation and other abiotic stresses in Brassica napus
Source: PLoS One. 2019 Jul 25;14(7):e0220374. doi: 10.1371/journal.pone.0220374 (PMC6657917; doi:10.1371/journal.pone.0220374)
Supplement: S5 Table — (DOCX) [file pone.0220374.s011.docx]

**S5 Table. Number of cis-elements in the 2.0-kb promoter regions of the PHT1 family genes in *Brassica napus.***

| **Gene name** | **Phosphorus homeostasis** | | **NaCl stress** | | **Drought stress** | | **Auxin-related** | | |
| --- | --- | --- | --- | --- | --- | --- | --- | --- | --- |
|  | **P1BS** | **W-box** | **GT-1** | **DRE** | **MBS** | **MYBR** | **TGA** | **CATATGGMSAVR** | **TATC-BOX** |
|  | **GNATATNC** | **TTGACC** | **GAAAAA** | **CCGA** | **TAACTG** | **TGGTTAG** | **AACGAC** |  | **TATCCCA** |
| *BnaPT1* | 3 |  | 8 |  |  |  |  |  |  |
| *BnaPT2* | 1 |  | 3 | 1 |  |  |  |  |  |
| *BnaPT3* | 1 |  | 1 | 2 |  |  |  | 1 |  |
| *BnaPT4* |  | 2 |  | 5 | 2 |  |  |  |  |
| *BnaPT5* | 1 | 1 | 3 | 1 | 2 |  |  |  |  |
| *BnaPT6* | 2 | 1 | 3 | 1 | 1 |  | 1 | 2 |  |
| *BnaPT7* | 1 |  | 2 | 2 |  | 1 |  | 1 | 1 |
| *BnaPT8* | 2 |  | 3 | 4 | 2 |  |  |  | 1 |
| *BnaPT9* |  |  | 5 | 1 |  |  |  |  |  |
| *BnaPT10* | 2 |  | 3 | 1 | 1 |  |  |  |  |
| *BnaPT11* | 3 | 1 | 2 | 1 | 1 |  |  |  |  |
| *BnaPT12* | 1 |  | 2 | 6 |  |  |  | 1 |  |
| *BnaPT13* | 1 | 1 | 1 | 7 |  |  |  |  |  |
| *BnaPT14* | 3 | 1 | 3 |  | 1 |  |  |  |  |
| *BnaPT15* | 3 |  | 1 | 1 |  |  | 2 |  |  |
| *BnaPT16* |  |  | 4 | 3 |  |  |  | 1 |  |
| *BnaPT17* | 2 |  | 1 | 5 |  | 1 |  |  |  |
| *BnaPT18* | 1 | 1 | 3 | 2 |  |  |  | 1 |  |
| *BnaPT19* | 1 |  | 2 | 4 |  | 1 | 1 | 1 |  |
| *BnaPT20* | 2 |  | 4 | 3 |  |  |  | 1 |  |
| *BnaPT21* |  |  |  |  |  |  |  |  |  |
| *BnaPT22* | 1 |  |  | 1 |  |  |  | 1 |  |
| *BnaPT23* | 1 |  | 2 | 1 |  |  | 2 |  |  |
| *BnaPT24* | 1 |  | 2 | 2 | 1 |  |  |  |  |
| *BnaPT25* | 1 |  | 2 | 3 |  |  |  | 1 |  |
| *BnaPT26* | 3 |  | 1 | 2 |  |  | 1 | 1 |  |
| *BnaPT27* | 2 | 1 | 3 | 5 | 1 |  | 2 |  |  |
| *BnaPT28* | 2 |  | 3 | 2 |  |  |  |  |  |
| *BnaPT29* |  |  | 6 | 1 |  |  |  | 2 |  |
| *BnaPT30* | 1 |  | 1 | 1 |  |  |  |  |  |
| *BnaPT31* |  | 2 |  | 5 | 2 |  |  |  |  |
| *BnaPT32* | 1 | 2 | 3 | 1 | 1 |  |  | 1 |  |
| *BnaPT33* | 1 | 1 | 6 | 1 | 2 |  |  |  | 1 |
| *BnaPT34* |  |  | 3 | 1 | 1 |  |  |  | 1 |
| *BnaPT35* | 2 | 1 | 1 | 1 | 1 |  |  |  |  |
| *BnaPT36* | 2 | 1 | 6 | 2 | 1 |  |  | 2 |  |
| *BnaPT37* | 2 |  | 3 | 3 | 1 |  |  |  | 1 |
| *BnaPT38* |  |  | 1 | 11 | 1 |  |  |  |  |
| *BnaPT39* |  |  | 4 | 3 |  |  |  |  |  |
| *BnaPT40* | 1 |  | 2 | 5 |  |  |  | 1 | 1 |
| *BnaPT41* | 2 |  | 4 | 4 | 1 |  |  | 2 |  |
| *BnaPT42* | 2 |  | 3 | 4 |  |  | 1 |  |  |
| *BnaPT43* | 1 |  | 4 | 1 |  |  | 1 | 1 |  |
| *BnaPT44* | 1 |  |  | 2 |  |  |  |  |  |
| *BnaPT45* |  |  | 1 | 1 | 1 |  | 1 |  |  |
| *BnaPT46* | 1 | 1 | 2 | 2 |  |  | 1 |  | 1 |
| *BnaPT47* | 3 | 1 | 4 | 3 |  |  |  | 1 |  |
| *BnaPT48* | 1 |  | 2 | 6 | 1 |  |  | 1 |  |
| *BnaPT49* | 2 |  | 4 | 1 |  |  |  | 1 |  |
